# Targeted Nano-Drug Delivery of Colchicine against Colon Cancer Cells by Means of Mesoporous Silica Nanoparticles

**DOI:** 10.3390/cancers12010144

**Published:** 2020-01-07

**Authors:** Khaled AbouAitah, Heba A. Hassan, Anna Swiderska-Sroda, Lamiaa Gohar, Olfat G. Shaker, Jacek Wojnarowicz, Agnieszka Opalinska, Julita Smalc-Koziorowska, Stanislaw Gierlotka, Witold Lojkowski

**Affiliations:** 1 Laboratory of Nanostructures, Institute of High Pressure Physics, Polish Academy of Sciences, Sokolowska 29/37, 01-142 Warsaw, Poland; a.swiderska-sroda@labnano.pl (A.S.-S.); j.wojnarowicz@labnano.pl (J.W.); a.opalinska@labnano.pl (A.O.); xray@unipress.waw.pl (S.G.); w.lojkowski@labnano.pl (W.L.); 2 Medicinal and Aromatic Plants Research Department, Pharmaceutical and Drug Industries Research Division, National Research Centre (NRC), Dokki, Giza 12622, Egypt; 3 Therapeutic Chemistry Department, Pharmaceutical and Drug Industries Research Division, National Research Centre (NRC), Dokki, Giza 12622, Egypt; hassan_heba59@yahoo.com; 4 Pharmacognosy Department, Pharmaceutical and Drug Industries Research Division, National Research Centre (NRC), Dokki, Giza 12622, Egypt; la_gohar@hotmail.com; 5 Medical Biochemistry and Molecular Biology Department, Faculty of Medicine, Cairo University, Cairo 11511, Egypt; olfatshaker@yahoo.com; 6 Laboratory of Semiconductor Characterization, Institute of High Pressure Physics, Polish Academy of Sciences, Sokolowska 29/37, 01-142 Warsaw, Poland; julita@unipress.waw.pl

**Keywords:** colchicine alkaloid, colon cancer cells, mesoporous silica nanoparticles, targeted delivery system, apoptosis, PD-1 immune checkpoint inhibitor and cancer immunotherapy

## Abstract

Antimitotics are important anticancer agents and include the natural alkaloid prodrug colchicine (COL). However, a major challenge of using COL as an anticancer drug is its cytotoxicity. We developed a novel drug delivery system (DDS) for COL using mesoporous silica nanoparticles (MSNs). The MSNs were functionalized with phosphonate groups, loaded with COL, and coated with folic acid chitosan-glycine complex. The resulting nanoformulation, called MSNsPCOL/CG-FA, was tested for action against cancer and normal cell lines. The anticancer effect was highly enhanced for MSNsPCOL/CG-FA compared to COL. In the case of HCT116 cells, 100% inhibition was achieved. The efficiency of MSNsPCOL/CG-FA ranked in this order: HCT116 (colon cancer) > HepG2 (liver cancer) > PC3 (prostate cancer). MSNsPCOL/CG-FA exhibited low cytotoxicity (4%) compared to COL (~60%) in BJ1 normal cells. The mechanism of action was studied in detail for HCT116 cells and found to be primarily intrinsic apoptosis caused by an enhanced antimitotic effect. Furthermore, a contribution of genetic regulation (metastasis-associated lung adenocarcinoma transcript 1 (MALAT 1), and microRNA (mir-205)) and immunotherapy effects (angiopoietin-2 (Ang-2 protein) and programmed cell death protein 1 (PD-1) was found. Therefore, this study shows enhanced anticancer effects and reduced cytotoxicity of COL with targeted delivery compared to free COL and is a novel method of developing cancer immunotherapy using a low-cost small-molecule natural prodrug.

## 1. Introduction

Colchicine (COL) is a natural alkaloid compound derived mainly from the medicinal plant Colchicum automnale and has been used in the clinic for treating gout and familial Mediterranean fever. COL has also shown some benefit in primary biliary cirrhosis [1], amyloidosis [2], and condyloma acuminate treatments [3]. COL is an antimitotic drug, which interferes strongly with cell division by affecting microtubule assembly and disassembly during mitosis. Most of the antimitotic drugs are toxic anticancer agents, which preferentially kill cancer cells, as they divide much faster than normal cells. However, the major challenge for COL is its toxicity, which causes severe side effects to patients [4].

Despite COL not yet being used clinically for cancer therapy because of its toxicity to normal cells, it is used as a lead compound in generating potent anticancer agents [5,6,7,8,9]. To reduce the side effects of COL on normal cells, many types of research have included analogs of COL [10,11] and combination treatments with other drugs [12]. Regarding the mechanism underlying the antimitotic effects of COL, several actions are associated with antimitotic and post-antimitotic responses. Gupta and Dudani [13] proposed that the mechanism of action for antimitotic drugs, such as COL, includes blockade of cell growth at metaphase upon the binding of antimitotic drugs to tubulin (for COL through the colchicine binding site) due to cell-cycle arrest (e.g., at G2/M). Following this action, the microtubules cannot exert any cellular functions [13]. In the presence of antimitotic drugs, cells either die during mitosis or exit mitosis. When they exit mitosis, several post-mitotic responses occur that can lead to cell death, including cell-cycle arrest and apoptosis [14]. In a report on the mitotic cell death that occurs during mitosis, Castedo et al. [15] proposed that the mitotic cell death catastrophe results from a combination of deficient cell-cycle checkpoints and cellular damage. Cell death occurring during mitosis is characterized by activation of caspase-2 in response to DNA damage, or caspase-9, caspase-3, and cytochrome c in response to mitochondrial membrane permeabilization. These effectors make up the molecular hallmarks of apoptosis. Thus, mitotic catastrophe is controlled by molecular players, including cell cycle-specific kinases, cell-cycle checkpoint proteins, caspases, and some proteins of the Bcl-2 family (e.g., Bax, Bcl-2), among others. Qi et al. provided evidence that, by arresting cell cycle progression in the presence of antimitotic drugs, mitotic spindles are disrupted, and cancer cells directly undergo apoptosis via the mitotic catastrophe [16].

Recently, antimitotic agents such as COL were reported to have a regulatory effect on most immune cell types, leading to the development of effective cancer immunotherapies [17]. With respect to cancer immunotherapy, targeting programmed cell death protein 1 (PD-1) and programmed cell death-ligand 1 (PD-L1), among other immune checkpoints, is important. PD-1 is an inducible immune modulatory receptor expressed on surface-activated T cells, and its ligand PD-L1 is expressed on cancer cells [18]. Binding PD-1 to PD-L1 leads to prevent from the immune antitumor effects by T cells against cancer cells [19]. However, recent studies have revealed the intrinsic expression of PD-1 in many cancers, along with immune T cells [20,21]. Therefore, it is considered a new potential target for cancer immunotherapy [19,21]. Thus, checkpoint blockade immunotherapy has revolutionized treatment for many tumors. In the next few years, scientists will be able to focus on immunotherapy research and broaden target cancers with different strategies [22]. Recently, the regulatory action by which antimitotic drugs inhibit PD-1 over-expression on the surface of T-cells was highlighted, and that PD-1 is strongly inhibited in the presence of the COL-binding site (CBS) of tubulin [23].

Traditional treatments for cancer, including surgery, radiation, cryosurgery, and chemotherapy, can be used alone or in combination. These methods have several limitations, such as toxicity, side effects, and expense. When chemotherapy is considered, normal treatment protocols include anticancer drugs alone or in combination to inhibit/kill cancer cells by affecting cell division and proliferation through different mechanisms. Drugs can produce various side effects for patients (e.g., neutropenia, liver and gastrointestinal toxicity, anemia, mucositis, and others) [24]. Therefore, cancer-targeting delivery systems with different nano-platforms have gained attention [25,26,27].

Despite the promising anticancer effects of COL, a few COL drug delivery systems (DDSs) have been tested to improve its therapeutic efficiency through cancer-targeting [28,29]. We focused on developing a novel DDS for COL by facilitating the active targeting of cancer cells. In the current study, we constructed a DDS for COL using three-dimensional fibrous dendritic mesoporous silica nanoparticles with a spherical shape (MSNs) known as KCC-1 type [26,30]. MSNs have been investigated as a drug delivery carrier for several drugs and biomolecules [27,31,32]. The tailored DDS comprises MSNs functionalized with phosphonate functional groups (MSNsP), and then loaded with COL MSNsPCOL, with the latter product subsequently coated with chitosan-glycine conjugated to folic acid (FA) to obtain a nanoformulation called MSNsPCOL/CG-FA. Glycine was employed as a source of amino groups that require for cancer growth [33]. FA was employed as the main targeting ligand, which is known for its binding potential to folate receptors, which are over-expressed on many cancers and facilitate the endocytosis pathway [26,34,35,36].

## 2. Results and Discussion

### 2.1. Synthesis and Characterization of the Targeted Delivery System

**Figure 1:**
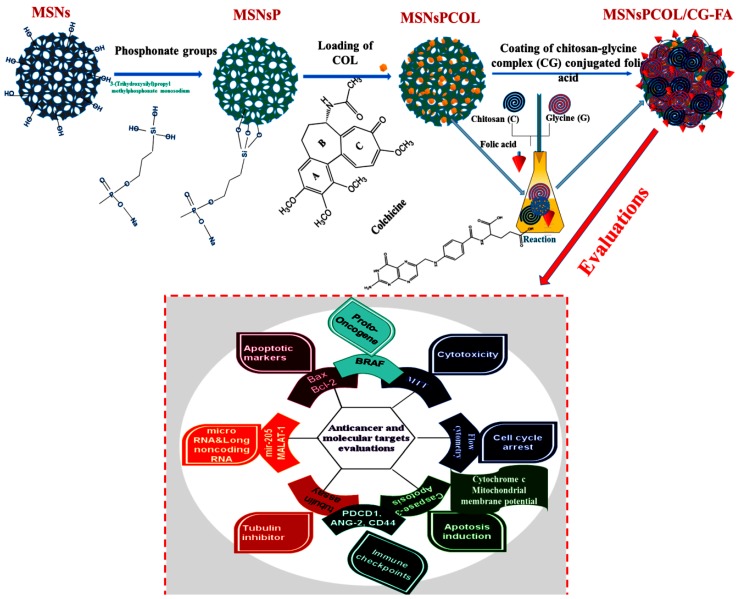
Schematic representation of the steps to obtain the proposed drug delivery system with the final product (MSNsPCOL/CG-FA) and various biological evaluations in vitro.

The targeted drug delivery system for COL, MSNsPCOL/CG-FA, comprised COL-loaded MSNsP subsequently coated with chitosan-glycine conjugated to folic acid. The schematic representation of the process is shown in Figure 1.

### 2.2. Electron Microscopy of Materials

**Figure 2:**
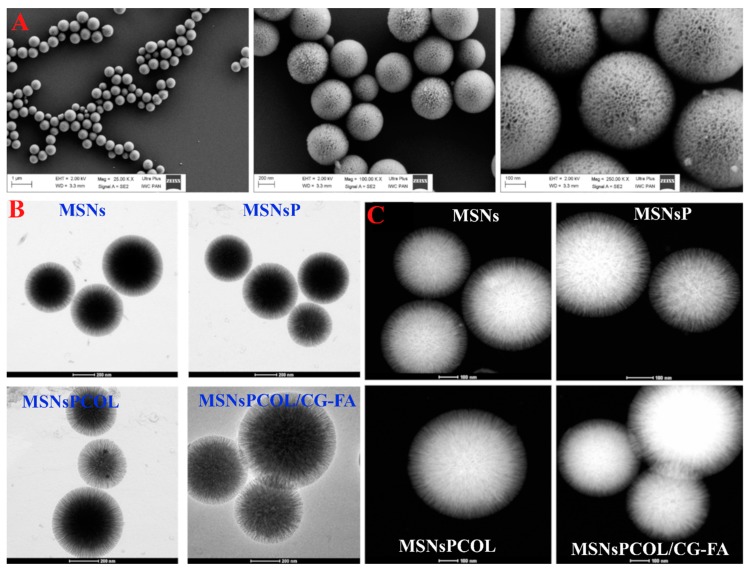
Morphological structures of the materials. (**A**) Field emission scanning electron microscopy (FE-SEM) of prepared mesoporous silica nanoparticles (MSNs) at different magnifications. (**B**) High-resolution transmission electron microscopy (HR-TEM) of prepared materials at different stages: Before and after of modification, colchicine (COL) loading, and coating. (**C**) Scanning transmission electron microscopy (STEM) of prepared materials at different stages: before and after of modification, COL loading, and coating.

To observe the structural changes between the prepared materials, we used TEM STEM and FE-SEM techniques. The FE-SEM images (Figure 2A) show the 3D dendritic mesoporous structure of MSNs with a spherical shape that is uniform in size. No aggregation was seen. In TEM images (Figure 2B), there were no detectable differences between MSNs, MSNsP, and MSNsPCOL. However, in MSNsPCOL/CG-FA, the central part appeared gray-white in color due to the coating complex (chitosan-glycine). This observation is similar to that seen in STEM images, where MSNsPCOL/CG-FA was white in color (Figure 2C). Thus, the coating was confirmed by STEM and TEM observations.

### 2.3. Surface Area Characteristics

The specific surface area and pore volume characteristics were measured (Table 1). Via modification with phosphonates, COL loading, and coating with CG-FA, both surface area and total pore volume were decreased compared to MSNs (380.1 m^2^/g and 0.772 cm^3^/g of specific surface area and total pore volume, respectively). This observation indicates successful preparation, in agreement with previous results [26,27,37,38].

### 2.4. Elemental Content Analysis

Energy-dispersive X-ray spectroscopy allowed us to determine the changes in the elemental content of prepared materials (Figure S1A, Supporting Information). Prior to modification, MSNs were composed of 53.18 wt% Si and 46.82 wt% O. Additional modification with organic phosphonate groups changed the elemental content, resulting in the presence of new elements, including 0.24 wt% P, 7.40 wt% C, and 0.95 wt% Na, as seen for MSNsP, confirming the modification. With further loading of COL on MSNsP, the C content increased and P content decreased in MSNsPCOL as a result of COL loading because it is an organic compound. Further polymer coating of MSNsPCOL relatively increased the C amount to 9.92 wt% and N amount to 2.77 wt% in MSNsPCOL/CG-FA. This observation is expected because both chitosan and glycine are composed of amino groups, which is important for confirmation of the coating process.

### 2.5. Particle Size Measurement

The particle size distribution was measured by nanoparticle tracking analysis (NTA) in aqueous solution (Figure S1B, Supporting Information). The mean size distribution for MSNs was 324 ± 33.2 nm. For MSNsP, the size increased to 407 ± 13.9 nm. For MSNsPCOL, the size slightly decreased to 391 ± 3.9 nm but was an insignificant difference. Unexpectedly, MSNsPCOL/CG-FA size decreased to 330 ± 22.2 nm compared to MSNsPCOL. An explanation may be the coating used for most of the particles with a smaller size compared to particles with a larger size, leading to an increase in their average size in the sample. This observation is in agreement with the two peaks appearing at 10–200 nm, which has not been observed previously.

### 2.6. Functional Group Determination

**Figure 3:**
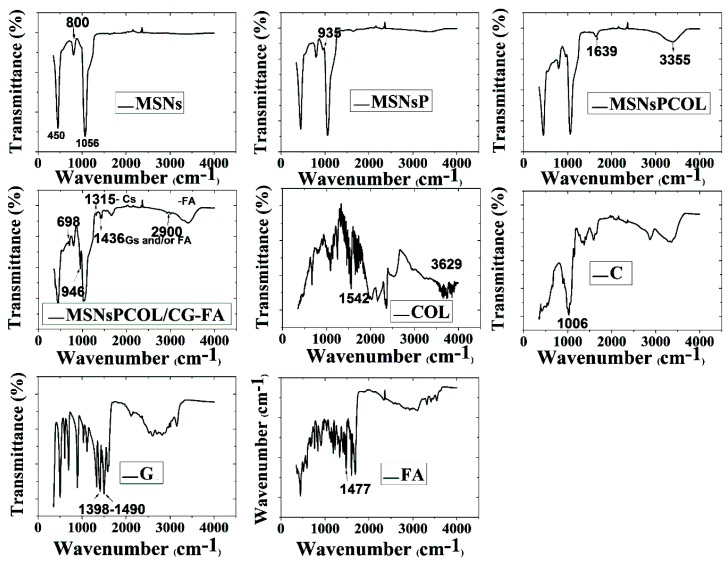
Fourier transform infrared spectroscopy (FTIR) spectra of MSNs before and after modification and COL loading, as well as chitosan (C), and glycine (G), folic acid (FA), and COL.

FTIR was used to identify surface functional groups (Figure 3). MSNs had several peaks at 450, 800, and 1056 cm^−1^ because of the siliceous mesostructured framework. As their surface was functionalized with phosphonate groups in MSNsP, we observed a new band at 953 cm^−1^ and a broad peak from 3000 to 3600 cm^−1^, reflecting phosphonate groups [39]. Two new peaks at 1639 cm^−1^, and an intensive peak centered at 3355 cm^−1^ were presented in MSNsPCOL, corresponding to shifted peaks at 1542 and 3629 cm^−1^ for COL. Additional coating resulted in several new peaks, with bands at 698 and 946 cm^−1^ attributed to chitosan, glycine, or folic acid. A band at 1315 cm^−1^ could correspond to chitosan, a band at 1436 cm^−1^ could correspond to either chitosan or folic acid, but a band at 2900 cm^−1^ could correspond to folic acid. A peak centered at 1034 cm^−1^ became broader, which ascribes all components for coating.

### 2.7. Simultaneous Thermal Analysis (STA-DSC) and XRD Characterization

**Figure 4:**
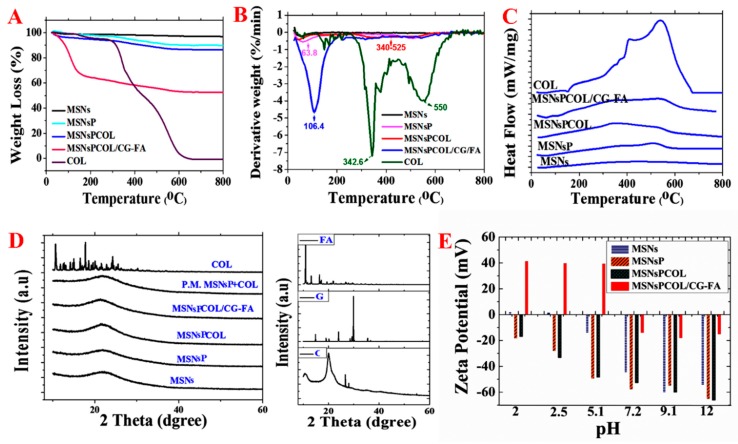
Thermal analysis of materials. (**A**) Simultaneous thermal analysis (STA) before and after surface modification, COL loading, and coating. (**B**) DTG analysis before and after surface modification, COL loading, and coating. (**C**) Differential scanning calorimetry (DSC) analysis before and after surface modification, COL loading, and coating. (**D**) X-ray diffraction (XRD) analysis before and after surface modification, COL loading, and coating. (**E**) Zeta potential measurements in aqueous solution before and after surface modification, COL loading, and coating.

To identify the mass fraction of phosphonate groups, COL, and polymer, thermal analysis was performed by calculating the weight loss values over their thermal decomposition (Figure 4A and Table 1). MSNs lost ~3.32 wt% because of moisture content. The weight loss further increased with MSNsP resulting from the decomposition of phosphonate groups. The heating of MSNsPCOL resulted in increased loss compared to MSNsP, which was calculated as COL loaded on MSNsP (3.60 wt%). The highest weight loss was recorded for MSNsPCOL/CG-FA, which was the result of decomposition of the coating complex (calculated to be 33.4 wt%), which confirms successful fabrication of the coating.

The results of the DTG thermograms in Figure 4B are consistent with STA, providing clear data that confirm the degradation. In MSNsP, a peak at 63 °C showed moisture, and another at 500 °C was attributed to organic decomposition. The degradation of COL was confirmed with the peak in the range of 340 to 525 °C in MSNsPCOL based on the two maximum peaks at 342 °C and 550 °C for free COL. A peak recorded at 106 °C in MSNsPCOL/CG-FA corresponded to decomposition of the coating material.

To further understand whether COL loaded on the surface of or inside particles, differential scanning calorimetry (DSC) analysis was conducted. Figure 4C shows no peaks in the MSNsPCOL and MSNsPCOL/CG-FA spectrum corresponding to free COL melting (409 to 537 °C). This observation shows that COL was enclosed in the particles.

To further verify the DSC results, we used XRD analysis. Figure 4D illustrates that no peaks appeared in the MSNsPCOL pattern corresponding to COL. We propose two reasons for this finding: Low loading and entrapment in pores. To confirm that we created a physical mixture of MSNsP and COL, no peaks were observed corresponding to COL. The MSNsPCOL/CG-FA pattern did not represent any peaks corresponding to chitosan, glycine, or folic acid, confirming the DSC data. These two complementary techniques show the characteristics of the meso-porosity of MSNs allowing the accommodation of various molecules, including functional groups, polymers, and drugs.

### 2.8. Zeta Potential Measurements

Figure 4E shows the differences in zeta potential for the materials and pH conditions. MSNs had a very low positive value (<+2 mV) in acidic media and very negative in alkaline medium (−55 mV). As expected, MSNsP had negative zeta values compared to MSNs associated with phosphonate groups. MSNsPCOL exhibited negative values at various pH values, probably due to the low COL content in particles. MSNsPCOL/CG-FA exhibited high positive values (>+40 mV) when medium was acidic (pH 2, 2.5 and 5.1). This observation is important for the cellular uptake of these particles by cancer cells to reach tumor sites. Negative values were found for MSNsPCOL/CG-FA particles in neutral or alkaline medium (pH 7.2, 9.9 and 12). The high positive zeta values for MSNsPCOL/CG-FA particles suggest that they can enter cells via endocytosis. The surface charge of particles (neutral, anionic, and cationic charges) determines their internalization into cells. Anionic nanoparticles are less efficiently internalized than cationic and neutral particles [40,41,42,43]. These results suggest that the developed DDS leads to higher cellular uptake in cancer cells.

### 2.9. MSN Cytotoxicity Evaluation

**Figure 5:**
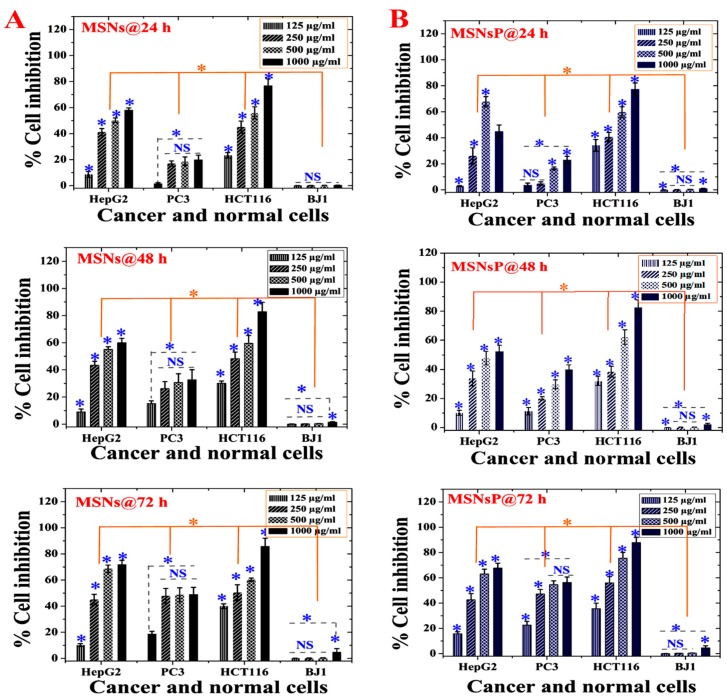
In vitro cytotoxicity (as percent inhibition) of MSNs and MSNs functionalized with phosphonate functional groups (MSNsP) for biocompatibility evaluations in cancer and normal cell lines after 24, 48, and 72 h of incubation with cancer cells (liver, HepG2; prostate, PC3; and colon, HCT116) and normal fibroblasts (BJ1). (**A**) Cytotoxicity of MSNs towards cell lines. (**B**) Cytotoxicity of MSNsP towards cell lines. Note: A blue asterisk (*) indicates significant (*p* < 0.05) differences between tested concentrations, whereas an orange asterisk (*) indicates significant differences between cell lines. NS, not significant. All data are expressed as mean ± SD.

Figure 5 presents the dependence of cell inhibition on concentration, time, cell line, and MSNs, with significant differences at *p* < 0.5. A gradual cell inhibition effect was found only when cells were treated with either MSNs or MSNsP at an increased concentration of 1000 µg/mL and incubation for 72 h. Higher cytotoxicity was recorded for HCT116 cells than PC3 and HepG2 cells, with 1000 µg/mL MSN and MSNsP treatment of HCT116 cells resulting in 85.9 ± 6.0% and 77.4 ± 4.7% inhibition, respectively. In contrast, normal BJ1 cells were less inhibited than cancer cells under the same treatment conditions.

The toxicity differences between MSNs and MSNsP varied according to cell line in response to concentration and time (Table S1 in Supplementary Information). With the IC50 value, it is possible to identify the differences in cytotoxicity; MSNs had a more toxic effect on HepG2 and HCT116 cells after 48 h compared to other incubations. In contrast, MSNsP had a more toxic effect on HCT116 cells after 24 and 72 h compared to 48 h. In addition, HCT116 cells were more sensitive than other cancer cell lines. Both types of nanoparticles had nearly equal IC50 values in PC3 cells after 24 and 48 h. Negligible cytotoxicity (IC50 > 1000 µg/mL) was observed for normal BJ1 cells in response to both types of nanoparticles. The negligible cytotoxicity on BJ1 normal cells can be related to the low internalization of nanoparticles in BJ1 normal cells. There is evidence in literature that cancer cells allow higher nanoparticles internalization compared normal cells due to the enhanced permeation and retention effect [44]. This, because of the vasculature of tumors, is often leaky, leading to accumulating nanoparticles in the bloodstream compared to normal tissue [45]. This finding agrees with previously published data for MCF-7 cells and BJ cells treated with MSNs and phosphonate-functionalized MSNs [39]. They mentioned that cancer cells uptake more MSNs than normal cells, and MSNs are more cytotoxic for cancer cells compared normal cells. Therefore, either MSNs or MSNsP is a promising nanocarrier for COL delivery.

### 2.10. In Vitro Anticancer Effects against Cancer Cells

**Figure 6:**
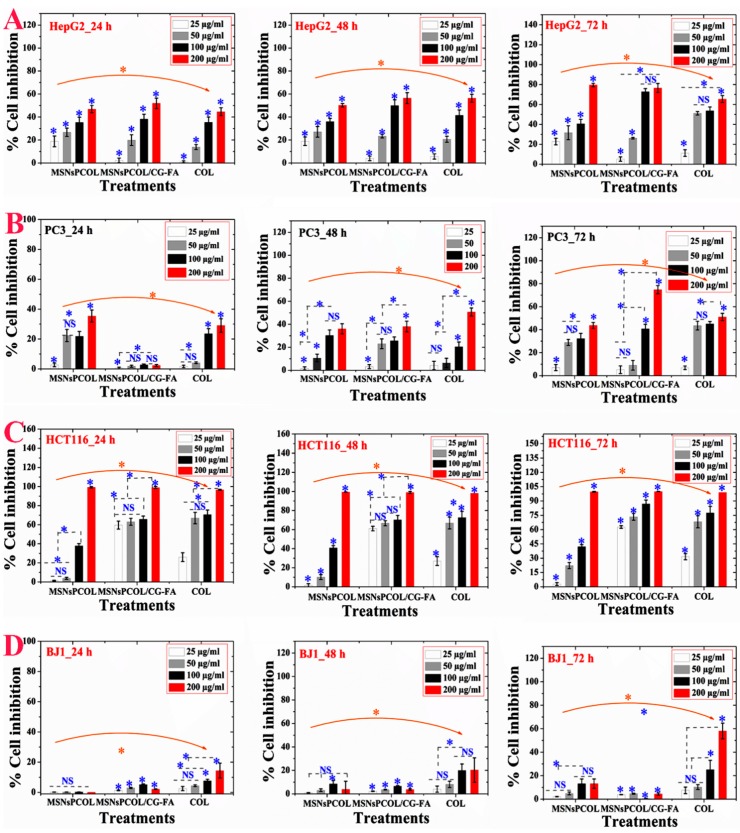
In vitro cytotoxicity (as percent inhibition) of the proposed delivery system in cancer and normal cells after 24, 48, and 72 h of incubation with cells. (**A**) Anticancer effects on HepG2 cancer cells. (**B**) Anticancer effects on PC3 cancer cells. (**C**) Anticancer effects on HCT116 cancer cells. (**D**) Anticancer effects on BJ1 normal cells. Note: A blue asterisk (*) indicates significant (*p* < 0.05) differences between tested concentrations, whereas an orange asterisk (*) indicates significant differences between tested samples (nanoformulations and COL). NS, not significant. All data are expressed as mean ± SD.

We studied the anticancer activity in terms of cell inhibition and found that it was significantly dependent on the cell line, concentration, incubation time, and delivery method. For HepG2 cells (Figure 6A), high inhibition was observed after 72 h and 200 μg/mL of all treatments. Regarding the role of the delivery route, MSNsPCOL/CG-FA exhibited high inhibition (80–82%), especially at 100 and 200 μg/mL, compared to MSNsPCOL and COL. This finding was also confirmed by IC50 values, with lower values detected for three incubation times with MSNsPCOL/CG-FA (Table S1 in Supplementary Information). Obviously, these results indicate that the anticancer activity against HepG2 cells was ranked in the following order: MSNsPCOL/CG-FA > COL > MSNsPCOL/CG-FA.

As shown in Figure 6B, PC3 cells were significantly inhibited by increasing dose and incubation time. Notably, MSNsPCOL/CG-FA had a gradual inhibitory effect compared to carriers; it had lower inhibition at 24 h and increased after 72 h, with a maximum inhibition of 80% when treated at 200 μg/mL. The anticancer effect in PC3 cells was ranked in this order: MSNsPCOL/CG-FA > COL > MSNsPCOL. This effect was also confirmed by IC50 values (Table S1 in Supplementary Information), with lower values obtained for MSNsPCOL/CG-FA compared to other treatments.

Interestingly, HCT116 cancer cells were highly inhibited compared to HepG2 and PC3 (Figure 6C). We observed that at a high concentration of 200 μg/mL MSNsPCOL, MSNsPCOL/CG-FA, and COL inhibited 100% at 24, 48, and 72 h. However, differences were seen among the three treatments when the concentration was decreased. MSNsPCOL/CG-FA potentially inhibited HCT116 growth >90% after 72 h compared to other carriers. Clear differences were confirmed by IC50 calculations, as MSNsPCOL/CG-FA had lower values than MSNsPCOL and COL and reached 19.7 μg/mL after 24 h, 17.4 μg/mL after 48 h, and 17.0 μg/mL after 72 h. The anticancer activity pattern against HCT116 was ranked in this order: MSNsPCOL/CG-FA > COL > MSNsPCOL. Thus, the findings concerning anticancer effects in three cancer cell lines show that HCT116 cells were more sensitive than HepG2 and PC3 cancer cells. Therefore, we selected HCT116 cancer cells for further investigation in our study.

For normal BJ1 cells, we observed that the inhibitory effect depended on the concentration and delivery route (Figure 6D). MSNsPCOL/CG-FA inhibited 4% after 72 h, compared to 60% for COL, which indicates negligible effects. As seen in the IC50 values (Table S1 in Supplementary Information), the delivery COL in the nano or free form had IC50 values > 100 μg/mL. MSNsPCOL/CG-FA significantly inhibited all tested cancer cells lines than COL ranking in this order: HCT116 > HepG2 > PC3. Also, MSNsPCOL/CG-FA had less or negligible toxic effects on normal BJ1 cells versus cancer cells than COL. Thus, this effect is the most likely to be developed as a DDS for cancer therapy.

From the observations, the proposed delivery route has an enhanced anticancer effect with a longer duration to 72 h. The reason for this is that the anticancer effect is in response to COL release from nanoformulations because it is controlled by a coating that needs some time for degradation. The polymeric coating controls the release of guest molecules from MSNs depending on time, pH, and other factors [27,31,46]. The enhanced anticancer effects with MSNsPCOL/CG-FA compared to free COL supports a cancer-targeting effect. This is possible through the interaction of folate receptors in cells and folic acid in nanoparticles. The sensitivity of HCT116 cancer cells to MSNsPCOL/CG-FA compared to HepG2 and PC3 is because folate receptors are overexpressed among cancer cell types [36]. It was shown that expression levels of folate receptors vary depending on type of cancer and the colorectal cancers show higher level than liver and prostate cancers [47]. However, the anticancer effect of free COL or enhanced delivery route is in response to inherited antimitotic effects from COL.

### 2.11. Inhibition of Tubulin Activity in HCT116 Cancer Cells

**Figure 7:**
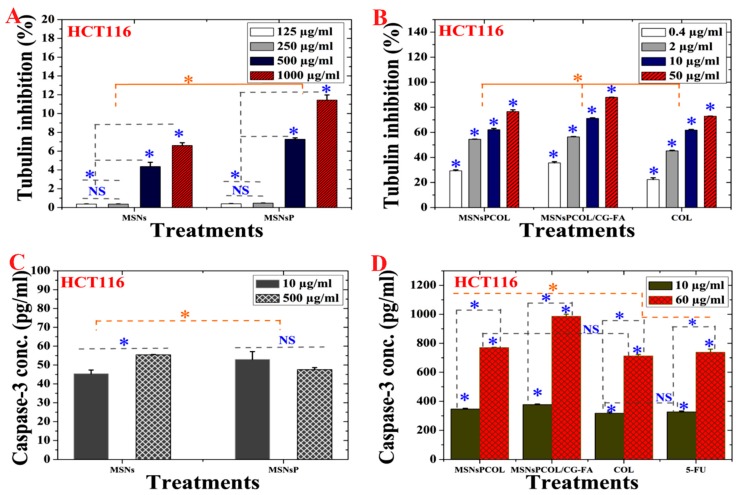
(**A**) Tubulin inhibition activity of HCT116 cells treated with MSNs and MSNsP as a function of concentration. (**B**) Tubulin inhibition activity HCT116 cells treated with the proposed delivery system as a function of concentration. (**C**) Caspase-3 activity of HCT116 cells treated with MSNs and MSNsP as a function of concentration. (**D**) Caspase-3 activity of HCT116 cells treated with the proposed delivery system and compared to the model anticancer drug (5-FU) as a function of concentration. Note: A blue asterisk (*) indicates significant (*p* < 0.05) differences between tested concentrations, whereas an orange asterisk (*) indicates significant differences between tested samples. NS, not significant. All data are expressed as mean ± SD.

As shown in Figure 7A, MSNs and MSNsP significantly inhibited tubulin in response to increasing the concentration used to treat cells. In this context, MSNsP inhibited ~12% compared to MSNs ~7% at 1000 μg/mL. Figure 7B shows that MSNsPCOL/CG-FA inhibited 90% more than COL at 50 μg/mL. Thus, the developed DDS is the most likely reason for enhanced tubulin inhibition because of the COL tubulin inhibitor agent [5,48,49]. We propose that the action for MSNsPCOL/CG-FA is the release of COL molecules into cells after folate receptor–folic acid interaction; the COL binds to tubulin in cells through colchicine binding sites, destabilizing tubulin [50], and further interferes with microtubule dynamics. The later response causes effective mitotic action against HCT116 cells. Further post-antimitotic response can arrest the cell cycle at the G2/M phase [51] and cause apoptotic cell death [52].

### 2.12. Cell Cycle Arrest at the G2/M Phase in HCT116 Cancer Cells

The G2/M checkpoint inhibitor class of drugs leads to DNA damage, preventing cells from passing mitosis and stopping their proliferation. As a result, cells cannot enter mitosis prior to repairing their DNA damage, resulting in apoptosis or death after their division [53] through various molecular signaling pathways [54,55]. Several reports have demonstrated that COL can arrest at G2/M in different cancer cells [7,56].

The cell cycle analysis was dependent on incubation time (Figure S2A,B in Supplementary Information). After 24 h, MSNsPCOL/CG-FA resulted in the maximum accumulation of cells at G2/M (14.8%), followed by 5-FU (12.4%), MSNsPCOL (11.2%), and COL (8.7%), with the lowest accumulation for MSNs (4.9%), MSNsP (4.8%), and untreated control (4.76%). In contrast, accumulation of cells at S phase was inhibited by MSNsPCOL/CG-FA (18.8%), MSNsPCOL (21.1%), 5-FU (21.2%), COL (23.3%), MSNsP (27.4%), and MSNs (27.6%) compared to control (27.7%). After 72 h, the number of cells increased at G2/M and deceased at S phase compared to 24 h. This cycle analysis pattern shows the endorsement of HCT116 cancer cell arrest at G2/M phase together with evidence that cells could not enter the S phase.

### 2.13. Enhancement of Apoptosis Induction in HCT116 Cancer Cells

**Figure 8:**
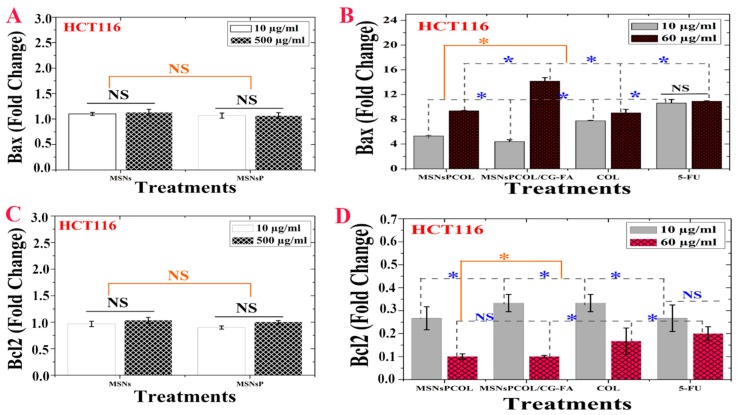
(**A**) Bax activity of HCT116 cells treated with MSNs and MSNsP as a function of concentration. (**B**) Bax activity of HCT116 cells treated with the proposed delivery system and compared to the model anticancer drug (5-FU) as a function of concentration. (**C**) Bcl-2 inhibition activity of HCT116 cells treated with MSNs and MSNsP as a function of concentration. (**D**) Bcl-2 inhibition activity of HCT116 cells treated with the proposed delivery system and compared to the model anticancer drug (5-FU) as a function of concentration. Note: A blue asterisk (*) indicates significant (*p* < 0.05) differences between tested concentrations, whereas an orange asterisk (*) indicates significant differences between tested samples. NS, not significant. All data are expressed as mean ± SD.

Apoptosis is a distinct or intrinsic occurrence relating to different physiological and pathological responses [55] and usually contributes to efficient antitumor action for most anticancer drugs [54,57]. To investigate whether the treatments induce apoptosis, HCT116 cells were stained with Annexin V-FITC and propidium iodide (PI), and then analyzed by flow cytometry. The parameters of late apoptosis, early apoptosis, and necrosis were quantified. Total apoptosis induction (early plus late) increased with treatment compared to untreated control cells after 72 h compared to 24 h (Figure S3A,B in Supplementary Information). The effects on total apoptosis were in this order: MSNsPCOL/CG-FA (15.0%) > MSNsPCOL (14.2%) > 5-FU (13.2%) > COL (10.6%) > MSNsP (0.5%) > MSNs (0.43). Compared to untreated control (0.5%), MSNs and MSNsP had no effects on apoptosis, reflecting the importance of DDS other than COL and 5-FU drugs. In addition to the induction of apoptosis, MSNsPCOL/CG-FA had a 1.4% necrosis effect. As MSNsPCOL/CG-FA promoted apoptosis (early and late apoptosis) (Figure 8B), the G2/M arrest triggers apoptosis in HCT116 cancer cells [58,59].

### 2.14. Activation of Caspase-3 in HCT116 Cancer Cells

The activation of intracellular caspase is one of the main characteristics of the apoptosis cell death pathway leading to the cleavage and inactivation of many cellular proteins, leading to the occurrence of apoptotic cell death in most cancer cell types [60]. As shown in Figure 7C, a significant effect was detected among MSNs and MSNsP. Treatment of cells with 500 μg/mL MSNs significantly increased activity compared to 10 μg/mL. In contrast, no significant differences were detected in either concentrations of MSNsP. Maximal activation of both treatments did not reach >55 pg/mL, which is a low enhancement effect. The caspase-3 activity was dependent on concentration and delivery method (Figure 7D), with high activity resulting from increased concentration to 60 μg/mL compared to 10 μg/mL. Maximal caspase-3 activity (>1000 pg/mL) was recorded for MSNsPCOL/CG-FA. Caspase-3 activation was enhanced in this order: MSNsPCOL/CG-FA > MSNsPCOL > 5-FU > COL. These results agree with our previous results using MSNs [26,27].

### 2.15. Modulation of Proapoptotic Bax/Bcl-2 in HCT116 Cancer Cells

Figure 8A shows no significant effect of MSNs and MSNsP on Bax, but these nanoparticles enhanced it to a little over 1 FLD. Figure 8B shows the significant difference for Bax at 60 μg/mL compared to 10 μg/mL. In addition, MSNsPCOL/CG-FA enhanced Bax to ~14 FLD, and the enhancement ranked in the following order: MSNsPCOL/CG-FA > 5-FU > COL > MSNsPCOL. In particular, MSNsPCOL/CG-FA strongly enhanced Bax compared to the clinically used 5-FU drug.

Though MSNs and MSNsP had not significant effect, they slightly inhibited BCL-2 ~1.2 FLD (Figure 8C). As shown in Figure 8D, the inhibition of BCL-2 was affected by concentration. Treatment of cells at 10 μg/mL resulted in low inhibition and ranked in effect as follows: MSNsPCOL and 5-FU (>0.2 FLD) > MSNsPCOL/CG-FA and COL (~0.35 FLD). Upon further increasing the concentration to 60 μg/mL, BCL-2 highly inhibited to ~0.1 FLD (for MSNsPCOL/CG-FA and MSNsPCOL), <0.2 FLD (for COL), and ~0.2 FLD. These findings show significantly better molecular targeting of nanoformulations in HCT116 cells via Bax enhancement and BCL-2 inhibition than free COL and 5-FU. Furthermore, this effect strongly requires efficient upregulation of Bax and down-regulation of BCL-2, allowing progression of the apoptotic pathway in HCT116 cancer cells. Inhibition of BCL-2 is important because it is considered a potent death suppressor protein; it promotes cell survival by blocking apoptosis and is upregulated in several cancers. BCL-2 is classified as an anti-apoptotic protein in many cancers, including colorectal cancer [61,62], inducing cancer resistance to drugs. Bax is a pro-apoptotic protein that promotes apoptosis in cells but is always present in inactivated states in many cancers, including colon cancer [57,63]. Therefore, these results confirm that the enhancement of apoptosis by MSNsPCOL/CG-FA interconnects with reduced Bcl-2 and increased Bax.

### 2.16. Inhibition of BRAF Expression in HCT116 Cancer Cells

Colon cancer is associated with multiple processes through various genetic alterations [64], including RAF genes, which mediate several cellular responses [65]. They contribute to carcinogenesis by upregulating the anti-apoptotic RAS/RAF/MEK/ERK pathway [66]. High expression of BRAF (anti-apoptotic protein) inhibits apoptosis by activating the BRAF/MEK/ERK route, which interferes with apoptosis through reduction of caspase and cytochrome c [67].

**Figure 9:**
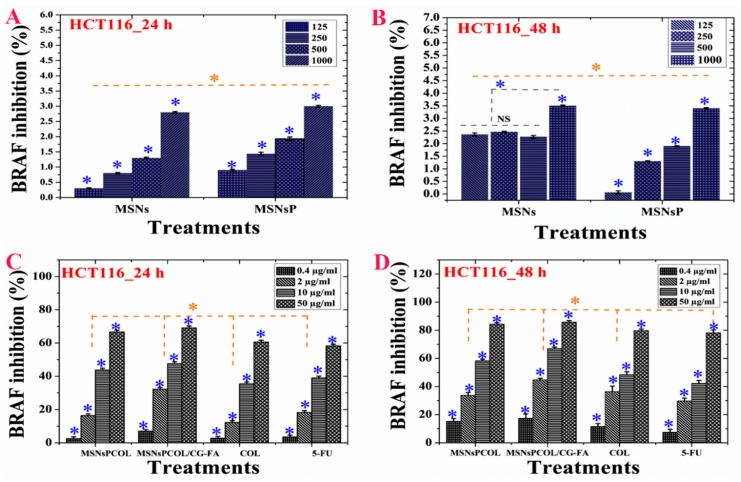
(**A**) BRAF activity of HCT116 cells treated with MSNs and MSNsP as a function of concentration. (**B**) BRAF activity of HCT116 cells treated with the proposed delivery system and compared to the model anticancer drug (5-FU) as a function of concentration. (**C**) BRAF inhibition activity of HCT116 cells treated with MSNs and MSNsP as a function of concentration. (**D**) BRAF inhibition activity of HCT116 cells treated with the proposed delivery system and compared to the model anticancer drug (5-FU) as a function of concentration. Note: A blue asterisk (*) indicates significant (*p* < 0.05) differences between tested concentrations, whereas an orange asterisk (*) indicates significant differences between tested samples. All data are expressed as mean ± SD.

BRAF inhibition was concentration- and time-dependent. MSNs and MSNsP (at 1000 µg/mL) reduced BRAF ~3% to 3.5% for cells incubated for 24 and 48 h, respectively (Figure 9A,B). Significant inhibition of BRAF was dependent on concentration, time, and delivery route (Figure 9C,D). It was decreased in cells incubated for a longer time. For example, at 50 µg/mL treatment, the inhibition was ~90%, ~87%, ~82%, and ~80% with MSNsPCOL/CG-FA, MSNsPCOL, COL, and 5-FU, respectively. This pattern shows that MSNsPCOL/CG-FA is efficient in inhibiting BRAF in HCT116 cells. Our findings on MSNsPCOL/CG-FA confirm the killing of HCT116 cancer cells was enhanced through apoptosis mechanisms.

### 2.17. Enhancement of Cytochrome c Triggers in HCT116 Cancer Cells

**Figure 10:**
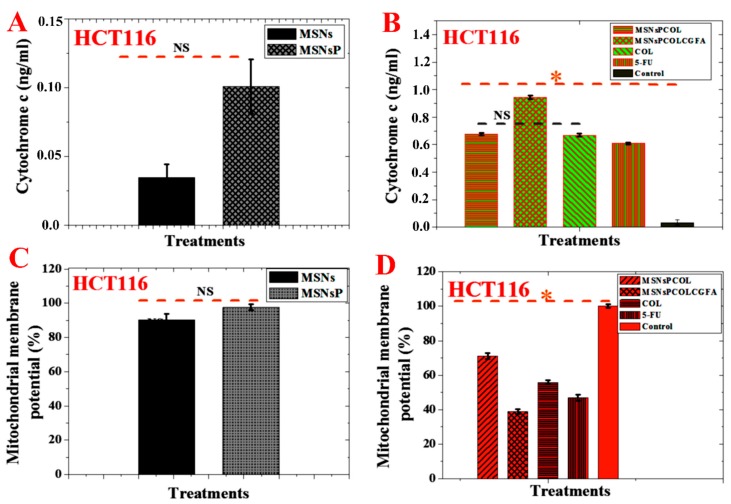
(**A**) Cytochrome c triggering of HCT116 cells treated with MSNs and MSNsP at 500 µg/mL. (**B**) Cytochrome c triggering of HCT116 cells treated at 60 µg/mL with the proposed delivery system for 72 h and compared to the model anticancer drug (5-FU) and control (without any treatment). (**C**) Mitochondrial membrane potential (Δψm) of HCT116 cells treated with MSNs and MSNsP at 500 µg/mL. (**D**) Mitochondrial membrane potential of HCT116 cells treated at 60 µg/mL with the proposed delivery system for 72 h and compared to the model anticancer drug (5-FU) and control. Note: An orange asterisk (*) indicates significant (*p* < 0.05) differences between tested samples. NS, not significant. All data are expressed as mean ± SD.

As shown in Figure 10A, no significant effect was detected for MSNs and MSNsP treated at 500 μg/mL for 72 h. Significant effect was detected in HCT116 cells treated with the nanoformulations compared to COL (Figure 10B). Triggering of cytochrome c was found in this order: MSNsPCOL/CG-FA > MSNsPCOL > COL > 5-FU. Our results agree with the results by Zhang et al. that COL triggers cytochrome c from the mitochondria to the cytoplasm in human gastric cancer cells [68]. These results confirm that the delivery system for COL activates apoptosis via the intrinsic apoptotic signaling pathway.

### 2.18. Reduction of Mitochondrial Membrane Potential in HCT116 Cancer Cells

No significant effect was observed among MSNs and MSNsP treated at 500 μg/mL for 72 h (Figure 10C). Importantly, treatment of HCT116 cells with MSNsPCOL/CG-FA significantly decreased mitochondrial membrane potential compared to MSNsPCOL, COL, 5-FU, and control (Figure 10D). Minimal mitochondrial membrane potential was found in this order: MSNsPCOL/CG-FA < 5-FU < COL < MSNsPCOL. It was reported that free COL decreased mitochondrial membrane potential in HT-29 colon cells, which led to intrinsic apoptotic cell death [6].

### 2.19. Inhibition of CD44 Expression in HCT116 Cancer Cells

As CD44 is a transmembrane glycoprotein, it can take part in different cellular processes, including growth, survival, cell differentiation, resistance to apoptosis [69,70,71], and tumorigenesis of colon cancer, such as HCT-116 cells [72]. CD44 is well-recognized as a robust marker (via overexpression) of colon cancer initiation because it promotes cell adhesion, maintains cell–matrix interactions [73], and induces anti-apoptotic properties [71,74]. Therefore, CD44 is a potential therapeutic target in colon cancer.

**Figure 11:**
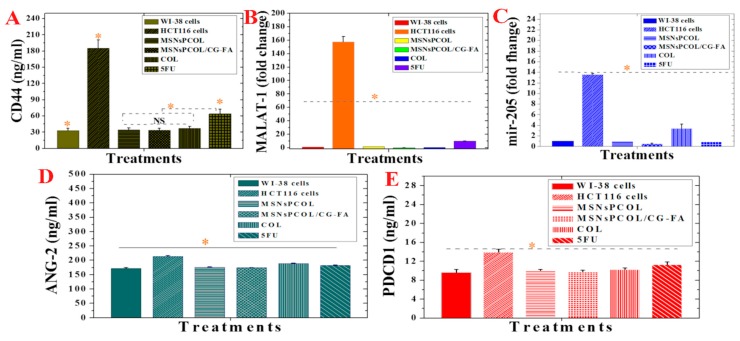
(**A**) MALAT-1 protein expression in normal WI-38 cells, HCT116 cells, and HCT116 cells treated with proposed delivery and 5-FU. (**B**) mir-205 expression in normal WI-38 cells, HCT116 cells, and HCT116 cells treated with proposed delivery and 5-FU. (**C**) Ang-2 protein expression in normal WI-38 cells, HCT116 cells, and HCT116 cells treated with proposed delivery and 5-FU. (**D**) CD44 protein expression in normal WI-38 cells, HCT116 cells, and HCT116 cells treated with proposed delivery and 5-FU. (**E**) PD-1 protein expression in normal WI-38 cells, HCT116 cells, and HCT116 cells treated with proposed delivery and 5-FU. Note: An orange asterisk (*) indicates significant (*p* < 0.05) differences between tested samples, whereas a blue asterisk (*) indicates significant differences between specific samples. NS, not significant. All data are expressed as mean ± SD.

MSNsPCOL/CG-FA, MSNsPCOL, and COL highly attenuated the CD44 concentration in HCT116 compared to positive control cells (185.5 ± 15.4 ng/mL; Figure 11A). We found no significant differences between MSNsPCOL/CG-FA (33.3 ± 3.5 ng/mL), MSNsPCOL (34.1 ± 3.3 ng/mL), and COL (37.3 ± 3.2 ng/mL). However, they significantly inhibited CD44 compared to 5-FU (63.7 ± 8.5 ng/mL). Concerning the anti-apoptotic effect of CD44 expression in colon cancer, Lakshman et al. previously reported that it prevents the apoptosis killing pathway because it promotes cell transformation into a malignant phenotype with the help of other anti-apoptotic factors in the tumor microenvironment [75]. We recorded the maximum inhibition for MSNsPCOL/CG-FA, supporting the results obtained for apoptosis induction. In a previous study of colon cancer cells, Park et al. reported that knockdown of CD44 leads to inhibition of cell proliferation and induction of apoptosis [71].

### 2.20. Inhibition of MALAT-1 Expression in HCT116 Cancer Cells

In recent years, emerging indications are that lncRNAs, non-protein coding transcripts longer than 200 nucleotides, are responsible for a broad spectrum of biological impacts with gene regulation and other functions in many diseases [76,77]. Among others, MALAT1 increases tumor formation in many cancers, including gastric, gallbladder, and lung cancer in vivo [77,78,79,80]. Importantly, MALAT1 has been shown in several studies to promote colorectal cancer cell development via proliferation, migration, and invasion [81,82], and is considered a potential therapeutic target for colon cancer. MALAT-1 was significantly inhibited by all delivery methods and free drugs (Figure 11B) compared to positive control cells. MSNsPCOL/CG-FA had a maximum inhibition effect with 0.3 ± 0.05-fold change, followed by COL (0.46 ± 0.05-fold change), MSNsPCOL (1.5 ± 0.15-fold change), and 5-FU (10.06 ± 0.57-fold change) compared to positive control cells (~160-fold change). Importantly, when HCT116 cells were treated with MSNsPCOL/CG-FA, the inhibition level was close to the level of normal cells. These results point out that COL and its nanoformulations are more efficient than 5-FU in hindering the expression of MALAT-1 in HCT116 cells, which is necessary for therapeutic targeting of colon cancer. To the best of our knowledge, no data are available yet on the effects of COL on MALAT1 inhibition or promotion.

### 2.21. Attenuation of mir-205 Expression in HCT116 Cancer Cells

Micro-RNAs are small non-coding RNAs discovered in 1993 that play crucial roles in cancer, including in cell viability, proliferation, invasion, metastasis, tumor suppressors, and oncogenes [83,84], allowing them to be used in cancer diagnosis and treatment [85,86]. Jing et al. showed that the expression of mir-205 is upregulated in plasma from colon cancer patients, permitting the occurrence and development of the cancer. Thus, mir-205 may be a potential tumor marker and therapeutic target [87]. However, mir-205 has also been reported to be downregulated in colon cancer [88].

Treating HCT116 cells with MSNsPCOL/CG-FA, MSNsPCOL, 5-FU, and COL decreased mir-205 expression. The inhibition values were 0.53 ± 0.05, 0.97 ± 0.05, 10 ± 0.3, and 3.4 ± 0.7-fold change, respectively, compared to the 14-fold change in positive control cells. This provides of evidence of possible mir-205 targeting in colon cancer (Figure 11C). To the best of our knowledge, there are no data available yet on the effects of COL in either downregulating or upregulating mir-205 in cancer. Our results may pave the way for further deep investigations.

### 2.22. Inhibition of Ang-2 Expression in HCT116 Cancer Cells

One characteristic of cancer is pathological angiogenesis, which plays a crucial role in selecting a therapy. Ang-2 is a pro-angiogenic cytokine that maintains angiogenesis and restricts the antitumor immune response from attacking cancer cells. In a study by Schmittnaegel et al., inhibition of Ang-2 together with vascular endothelial growth factor A (VEGFA) enhanced the antitumor immunity, allowing PD-1 checkpoint inhibition [89]. In addition, Kim et al. indicated that Ang-2 maybe play a crucial role as an oncogene in colorectal carcinogenesis, supporting tumor progression as a prognostic marker [90]. Therefore, we sought to explore the proposed DDS for the possibility of inhibiting Ang-2 and further explain the PD-1 checkpoint inhibitor.

A significant difference was detected between all treatments and positive control cells (HCT116 cells with no treatment; Figure 11D). Notably, treated HCT116 cells had strongly inhibited expression of Ang-2, which was slightly like its expression level in normal cells (172.1 ± 2.5). A maximum inhibition of Ang-2 recorded for MSNsPCOL/CG-FA (174.0 ± 0.75 ng/mL) > MSNsPCOL (175.7 ± 1.0 ng/mL) > 5-FU (182.3 ± 1.15 ng/mL) > COL (188.6 ± 1 ng/mL) compared to positive control cells (213.3 ± 3.0 ng/mL). These results suggest that the anticancer activity of the proposed DDS could promise to target Ang-2 supporting the immune checkpoints inhibition [89,91].

### 2.23. Inhibition of PD-1 Expression in HCT116 Cancer Cells

Figure 11E shows the suppression of PD-1 checkpoint with significant changes compared control cells; the maximum inhibition level was reached at 9.8 ± 0.3 ng/mL and 10 ± 0.3 ng/mL for MSNsPCOL/CG-FA and MSNsPCOL, respectively, compared to 14 ± 0.5 ng/mL with positive control cells. This was similar to the PD-1 concentration in normal BJ1 cells (9.6 ± 0.5), which is significant. PD-1 reached 10.3 ± 0.2 and 11.2 ± 0.5 with free COL and 5-FU, respectively.

Recent studies have revealed the intrinsic expression of PD-1 in many cancers, along with T immune cells [20,21]. Therefore, it is considered as a new potential for cancer therapy [19] and efficient treatments [92]. Checkpoint blockade immunotherapy has revolutionized treatment for tumors and, in the next years, scientists are expected to focus on immunotherapy research and broaden its scope to target cancers by different strategies [22]. PD-1 immune checkpoint inhibitors act by blocking the PD-1 protein and activate the immune system to treat many tumors [22,93]. MSNsPCOL/CG-FA inhibited PD-1 in HCT116 cells to the range close to normal BJ1 cells, which is important. These are the first results for COL or a DDS as a small drug molecule. PD-1 inhibitors are usually large molecules, such as antibodies, antigens, peptides, and therapeutic proteins. Although many approved therapies are efficient in cancer (e.g., nivolumab and pembrolizumab), they have many limitations, including side effects, toxicity, internal response changes in the immune system, and expense [94,95]. The results create an opportunity to develop a new approach of PD-1 inhibitors through further ongoing research.

**Figure 12:**
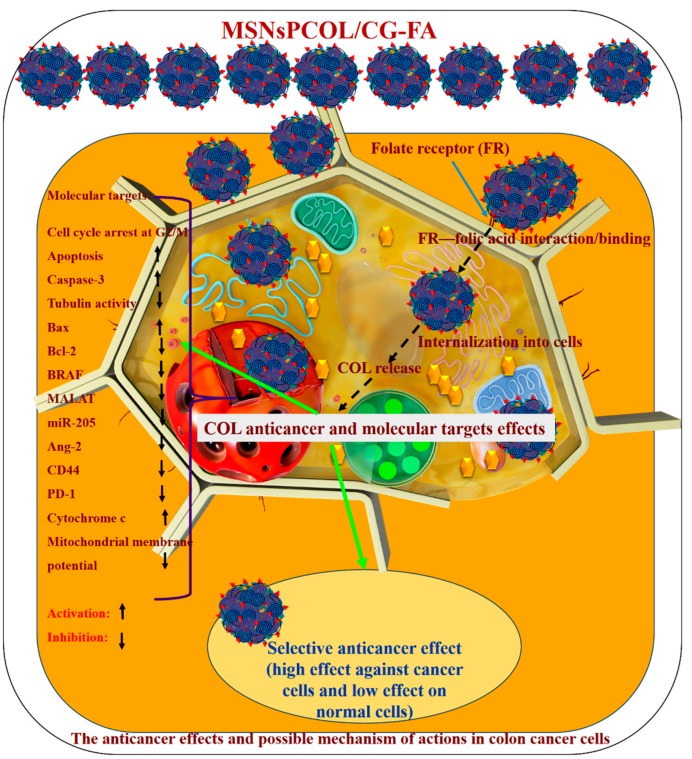
Schematic representation of cancer cell killing and possible anticancer mechanisms by which the proposed drug delivery system for colchicine (MSNsPCOL/CG-FA nanoformulation) acts in vitro against HCT116 colon cancer cells.

In summary, the main findings in the current study were enhancement of the antimitotic effects of COL by means of a targeted delivery system (MSNsPCOL/CG-FA). The enhanced antimitotic effects block cancer cell growth due to the binding of COL to tubulin in cells, leading to cell death. The main mechanism of action is an apoptosis cell death mechanism through different molecular pathways known for COL action on cancer cells. In addition, genetic regulation and immunotherapeutic effects have a role. A schematic representation of the nanoformulation mechanism of action is shown in Figure 12, and Table 2 outlines the results of the current study. The following factors explain the feasibility of cancer-targeting via folate receptors using folic acid ligands as developed here:

(1) The developed nano-delivery system efficiently targets the interaction of folic acid (on the surface of MSNsPCOL/CG-FA) with folate receptors over-expressed on cancer cells. In a next step, it releases COL into cancer cells, which can bind to the tubulin forming the microtubules in the cellular skeleton, resulting in tubulin inhibition. This effect is important because microtubules enable cells to undergo mitosis or subsequent intracellular post-antimitotic responses. Maximum tubulin inhibition (~90%) was achieved when HCT116 cancer cells were treated with MSNsPCOL/CG-FA.

(2) By producing the antimitotic effect via tubulin inhibition, the cells died. The cell-killing was cell line-, concentration-, time-, and delivery method-dependent. We observed that HCT116 cells were more sensitive to treatments than HepG2 and PC3 cells. In the case of HepG2 and PC3 cells, MSNsPCOL/CG-FA efficiently inhibited the cells compared to COL at all concentrations used. In the case of HCT116 colon cells, a strong effect was observed when incubating cells for 72 h. MSNsPCOL/CG-FA and COL had equal inhibition (100%) at 200 µg/mL. At lower concentrations, MSNsPCOL/CG-FA significantly inhibited HCT116 cells compared to COL. Thus, at all concentrations, MSNsPCOL/CG-FA was more efficient than COL in HCT116 cells. Concerning IC50, MSNsPCOL/CG-FA had a lower value (17.0 μg/mL) than free COL after 72 h. In addition, MSNsPCOL/CG-FA had less toxicity towards normal cells (10%) than COL (60%).

(3) Because COL inhibits tubulin, the post-antimitotic response resulted in various molecular pathways, including cell-cycle arrest, apoptosis, and genetic regulation [14]. We investigated several molecular, genetic, and immunology pathways to explore the mechanism of action. The results confirm that tubulin was markedly inhibited, especially with MSNsPCOL/CG-FA compared to COL. Because of the inhibition of tubulin and the resulting cell cycle arrest, we investigated cell cycle analysis by flow cytometry. MSNsPCOL/CG-FA enhanced the cell-cycle arrest at G2/M phase compared to COL. This observation confirms the tubulin inhibition effect. Arresting cells at the G2/M phase results in apoptosis. The maximum induction of apoptosis was detected for MSNsPCOL/CG-FA. Therefore, this observation confirms that the apoptosis cell death mechanism occurs in HCT116 cancer cells. To further confirm whether apoptosis is the main mechanism of action, we tested many molecular pathways. We observed that caspase-3 was enhanced when cells were treated with MSNsPCOL/CG-FA compared to other formulation, and this is one of the main routes for apoptosis. The modulation of pro-apoptotic proteins is shared with apoptosis; treating cells with MSNsPCOL/CG-FA promoted Bax and inhibited Bcl-2 protein. The anti-apoptotic BRAF protein and CD44 cellar protein levels were highly inhibited after cells were treated with MSNsPCOL/CG-FA. The observations provide strong evidence of the intrinsic apoptosis mechanism due to enhanced cytochrome c triggering and reduce the mitochondrial membrane potential, in agreement with the literature.

(4) New effects were obtained in our study. The antimitotic drugs act by inducing various genetic regulations that may or may not be related to the apoptosis mechanism. Our results show that MSNsPCOL/CG-FA inhibited MALAT 1 and mir-205 in treated cells. Regarding the importance of cancer immunotherapy because of the post-antimitotic effects, MSNsPCOL/CG-FA markedly inhibited Ang-2 protein and PD-1 in HCT116 cancer cells compared to normal WI-38 cells. To the best of our knowledge, the results for MALAT1, mir-205, and PD-1 with COL and its delivery in HCT116 cancer cells were obtained for the first time. The findings suggest that the killing of cancer cells is in response to the effects of the post-antimitotic response of the COL delivery route. We propose that the anticancer mechanism is mainly apoptosis cell death, with the contribution of genetic regulation and immunotherapy effects.

## 3. Materials and Methods

### 3.1. Materials

COL, 5-fluorouracil (5-FU), tetraethyl orthosilicate (TEOS), cetylpyridinium bromide (CPB), cyclohexane, isopropanol, urea, glycine, folic acid, insulin, penicillin G, streptomycin, and MTT assay kits were purchased from Sigma-Aldrich (St. Louis, MO, USA), and the Caspase-3 (active) Human ELISA kit from Invitrogen (Camarillo, CA, USA). 3-(Trihydroxysilyl)propyl methylphosphonate monosodium salt solution was purchased from Santa Cruz Biotechnology, Texas, USA. Methanol, ethanol, and acetic acid were obtained from Fisher Scientific UK (Loughborough, UK). Dimethyl sulfoxide (DMSO) was obtained from Tedia (Fairfield, OH, USA). 1-(3-dimethylaminopropyl)-3-ethylcarbodiimide hydrochloride (EDC), *N*-hydroxysuccinimide (NHS), and chitosan (100,000-300,000 Da) were obtained from Acros Organics (Geel, Belgium). Phosphate-buffered saline (PBS), Dulbecco’s modified Eagle medium (DMEM), Roswell Park Memorial Institute medium (RPMI 1640), and fetal bovine serum (FBS) were obtained from Gibco/Life Technologies (Thermo Fisher Scientific, Langenselbold, Germany). Insulin (Novo Nordisk, Bagsvaerd, Denmark) and trypsin versene (Vacsera, Giza, Egypt) were also used. The well plates were obtained from Greiner Bio-One GmbH (Frickenhausen, Germany). The Annexin V-FITC Apoptosis Detection Kit was from BioVision (Mountain View, CA, USA), the flow cytometry kit for cell cycle analysis (ab139418) from Abcam^®^ (Cambridge, UK), Script One-Step RT-PCR Kit with SYBR^®^ Green from BIO-RAD (Hercules, CA, USA), Human Angiopoietin 2 ELISA Kit and Human Programmed Cell Death Protein 1 ELISA Kit from Bioassay Technology Laboratory Systems (Shanghai, China), Human CD44 ELISA kit from Gen-Probe Diaclone SAS (Besançon, France), and the miRNeasy extraction kit from Qiagen (Valencia, CA, USA). Enzyme-linked immunosorbent assays (ELISAs) used a kit for tubulin β (TUBb; SEB870Hu, Cloud-Clone Corp., Houston, TX, USA) and human B-RAF/B-Raf sandwich ELISA (LifeSpan BioSciences, Seattle, WA, USA). TMRE Mitochondrial Membrane Potential Assay Kit (Cymans Chemical, Ann Arbor, MI, USA). Cytochrome c Human ELISA Kit (abcam, Austria). Ultrapure water (18.2 MΩ; Milli-Q^®^ system, Millipore, Darmstadt, Germany) was used in all prepared solutions. All other reagents were of analytical grade.

### 3.2. Methods

#### 3.2.1. Synthesis and Modification of Mesoporous Silica Nanoparticles

We prepared MSNs of KCC-1 type nanospheres with a 3D fibrous dendritic structure using the detailed synthesis methods according to Polshettiwar et al. [30] and AbouAitah et al. [26]. For further surface modification through the post-synthesis route, to graft phosphonate groups, the MSNs were dried at 50 °C for 5 h to remove the physically adsorbed water. Next, 1 g of dried MSNs was suspended in 100 mL of ultra-pure water with the aid of sonication (Elma GmbH, Singen, Germany) for 30 min, and then 1.5 mL of 3-(trihydroxysilyl)propyl methylphosphonate monosodium salt was added drop-wise over with stirring. The mixture solution was left at room temperature under reflux conditions for 24 h. The material was collected by centrifugation (Cooling Sigma 16K, Laborzentrifugen GmbH, Osterode am Harz, Germany) at 10,000 rpm for 10 min, and then washed three times with methanol. Finally, the material was dried in an oven at 50 °C for 6 h to obtain MSNsP.

#### 3.2.2. Colchicine Loading

To load COL into MSNsP, we followed our previous method with some modification [38]: 100 mg COL was dissolved in 10 mL deionized water (pH 7), and then 300 mg of MSNsP was added and stirred (DAIHAN Scientific, Seoul, Korea) for 24 h at room temperature. The solution was centrifuged (Cooling Sigma 16K, Laborzentrifugen GmbH, Osterode am Harz, Germany) for 10 min and washed with deionized water once. Finally, the collected material was heated in an oven at 50 °C for 12 h and labeled as MSNsPCOL.

#### 3.2.3. Coating and Conjugation with Chitosan-Glycine and Folic Acid

Coating with chitosan-glycine and conjugating with folic acid was achieved via the following steps. First, the chitosan-glycine was prepared based on a coacervate and EDC/NHS coupling reaction [27] by dissolving 250 mg of chitosan in 20 mL (2%) acetic acid and stirred for 2 h at 60 °C (solution A). In another beaker, 100 mg of glycine was dissolved in 10 deionized water, followed by the addition of 60 mg EDC and 50 mg NHS and stirring for 2 h at room temperature (solution B). Solution B was introduced to solution A drop-wise and left to stir for 4 h at 50 °C. Hereafter, the mixture solution is referred to as the CG complex solution. Second, the activation of folic acid was carried out by dissolving 85 mg of folic acid, 70 mg of EDC, and 50 mg of NHS in 20 mL DMSO, stirring the mixture for 20 h at room temperature. Third, the activated folic acid solution was added drop-wise into the CG complex solution and stirred for 4 h at 50 °C to obtain CG-FA complex solution. The solution was kept at −20 °C until further use. Fourth, COL-loaded nanoparticles were coated with chitosan-glycine and conjugated to folic acid by dispersing 600 mg of MSNsPCOL into 15 mL of CG-FA complex solution and stirring at room temperature for 24 h. The MSNsPCOL-CG-FA product was collected by centrifugation and washed with ethanol and ultra-pure water, then dried in an oven at 50 °C for 12 h.

#### 3.2.4. Characterization Techniques

The following techniques were used to characterize the obtained materials: Field emission scanning electron microscopy (FE-SEM; Ultra Plus, Zeiss, Germany) equipped with QUANTAX EDS (Bruker); scanning transmission electron microscopy (STEM; FEI TECNAI G2 F20 S-TWIN, Thermo Fisher Scientific, Waltham, MA, USA); powder X-ray diffraction (XRD; X’PertPRO System, PANalytical) using CuKα radiation in the 2θ range of 10–100; and Brunauer, Emmett, and Teller (BET) specific surface area analysis using a Gemini 2360 instrument (Micromeritics) according to ISO 9277:2010. Before the density and SSA measurements were carried out, the powders were dried at 150 °C (without drug) or 50 °C (with drug and polymer) for 24 h under a constant flow of helium (FlowPrep 060 desorption station by Micromeritics). Fourier transformed infrared (FTIR) spectroscopy (Bruker Optics Tensor 27, Bruker Corporation, Billerica, MA, USA) was performed with attenuated total reflectance (ATR, model Platinum ATR-Einheit A 255). Simultaneous thermal analysis (STA)-coupled differential scanning calorimetry (DSC) (STA-DSC) analysis was performed using the STA 499 F1Jupiter (NETZSCH-Feinmahltechnik GmbH, Germany). Samples weighing 10–20 mg were inserted into the alumina pan of the STA unit, and before measurements, helium was flowed through the STA furnace chamber for 30 min. The experimental parameters were programmed to reach 850 °C with a heating rate of 10 °C/min under a helium/air mixture. Zeta potential measurements using a Malvern ZetaSizer (NanoZS, UK) were performed based on the water suspension of nanoparticles at 24 °C. Water suspensions were used for nanoparticle tracking analysis (NTA) using NanoSight NS500 instrument (Malvern Instruments Ltd., Malvern, UK) and data analyzed by NanoSight software (Malvern Instruments Ltd., Malvern, UK).

#### 3.2.5. In Vitro Cytotoxicity Assessment

For the estimation of in vitro cytotoxic potency, the 3-4,5-dimethylthiazol-Z-yl-2,5-diphenyltetrazolium bromide (MTT) assay was conducted according to Mosmann [96]. Human prostate adenocarcinoma PC3 cells (ATCC^®^CRL-1435 ^TM^), human colon colorectal carcinoma HCT116 cells (ATCC^®^CCL-247^TM^), human hepatic carcinoma HepG2 cells (ATCC^®^ HB-8065^TM^), and human fibroblast BJ1 cells (ATCC^®^CRL-2522™) were grown in RPMI supplemented with 10% fetal bovine serum (Cologne, Germany) and 1% penicillin-streptomycin solution (penicillin 10,000 IU/mL; streptomycin 10,000 μg/mL. The cell lines were cultured at 37 °C in 95% humidity and 5% CO_2_, and subcultured twice weekly using trypsin versene 0.15%. The cells were seeded in flat-bottom 96-well plates at a density of 10,000 cells per well for 24 h. The serum enriched medium was used during the bioassay to keep the cells alive through the long duration of the bioassay and to better simulate in vivo conditions. Cells were treated with the different samples diluted in medium at 125, 250, 500, and 1000 µg/mL (for MSNs and MSNsP) to test the biocompatibility of nanoparticles. For anticancer activity, cells were treated (as equivalent amount to COL in nanoformulations, the equivalent amount used throughout all studies for investigated nanoformulations) at 25, 50, 100, 200 µg/mL (for MSNsPCOL, MSNsPCOL/CG-FA, COL, 5-FU). After treatment for 24, 48, or 72 h, 10 μL of MTT was added, and after 5 h of incubation the color was measured at 495 nm against the 690 nm reference. Finally, the percent cytotoxicity was calculated according to [1-(av(S)/(av(NC))] × 100, where av(NC) is the average absorbance of the three negative control wells measured at 495 nm (reference 690 nm) and av(S) is the average absorbance of the three sample wells measured at 495 nm (reference 690 nm). IC50 and IC90 values were calculated using Probit analysis and SPSS for Windows statistical analysis software package, version 9 (1989, SPSS Inc., Chicago, IL, USA).

#### 3.2.6. Apoptosis Detection and Cell Cycle Analysis with Flow Cytometry

To analyze apoptosis and the cell cycle, we followed manufacture protocols and our pervious study [27]; HCT116 cells were seeded on a six-well plate at a density of 2 × 10^5^ cells/per well in RPMI 1640 supplemented with 10% FBS, 1% penicillin/streptomycin and incubated at 37 °C in a 5% CO_2_ atmosphere. After 24 h, the medium was replaced with fresh, treated with different samples at 40 µg/mL (100 µL), and incubated for 24 h or 72 h. Control cells received no treatments. For cell cycle analysis, cells were trypsinized, washed with cold PBS, and fixed with 70% ethanol. The fixed cells were rinsed with PBS, and then labeled with propidium iodide (PI) following the manufacturer’s instructions (Abcam^®^, Cambridge, UK). Finally, cells were analyzed by flow cytometry (FACSCalibur, Becton Dickinson, NJ, USA). Cell cycle analysis was performed with an FL2-A histogram of single cells. The Annexin V-FITC Apoptosis Detection Kit (BioVision, CA, USA) was used to detect apoptosis according to the manufacturer’s instructions. After treating the cells as mentioned, the cells were trypsinized, fixed in 70% ethanol, washed with cold PBS, and suspended in binding buffer (500 µL). Next, 5 μL of Annexin V-FITC and 5 μL of PI were added and incubated for 10 min in the dark, and then immediately analyzed by flow cytometry (FACSCalibur, Becton Dickinson, NJ, USA).

#### 3.2.7. Caspase-3 Activity Assay

The caspase-3 activity was determined according to the manufacturer’s instructions using the human active caspase-3 content assay kit. HCT116 cells were cultured on 96-well plates (1.2–1.8 × 10,000 cells/well) in 100 µL of RPMI 1640 containing 10% FBS, 1% penicillin/streptomycin at 37 °C. The cells were treated at 10 and 500 µg/mL (for MSNs and MSNsP) or 10 and 60 µg/mL (for MSNsPCOL, MSNsPCOL/CG-FA, COL, 5-FU) with 100 μL sample volume per well and incubated for 72 h prior to the assay. The procedures are reported in detail in our previous study [27]. The absorbance was measured at 450 nm using the Robonik P2000 ELISA reader. The assay was performed in triplicate, and data are expressed as mean ± SD.

#### 3.2.8. Tubulin Assay

To assess tubulin polymerization, the ELISA kit for TUBb (SEB870Hu, Cloud-Clone Corp., Houston, TX, USA) was used following the manufacturer’s instructions. HCT116 cells were seeded in 96-well plates at a cell density of 1.2–1.8 × 10,000 cells/well in 100 µL growth medium (DMEM); 100 µL of each sample was added per well. MSNs and MSNsP were tested at 125, 250, 500, and 1000 µg/mL, whereas MSNsPCOL, MSNsPCOL/CG-FA, and COL were used at 0.4, 2, 10, and 50 µg/mL. After 48 h of incubation, the solution was removed, cells were detached by trypsinization, washed with cold PBS buffer, suspended in PBS, and followed by three freeze/thaw cycles to lyse the cells. Cell lysates were centrifuged in a cooling centrifuge for 10 min, which permits the removal of cellular debris to detect β-tubulin in the supernatant. The assay was performed as instructed in the kit. Finally, ELISA reader ROBONIK P2000 (Robonik India PVT LTD, Thane, India) was used to measure the color at 450 nm in triplicate. Data were calculated as percent inhibition.

#### 3.2.9. Expression of Bax and Bcl-2

##### Cell Culture Treatment and RNA Extraction

HCT116 cells were cultured at a density at 1 x 10^6^ and incubated for 48 h at 10 and 500 µL (for MSNs and MSNsP) or 10 and 60 µg/mL (for MSNsPCOL, MSNsPCOL/CG-FA, COL, and 5-FU). Cells were then collected for RNA extraction using the RNeasy extraction kit according to the manufacturer’s protocol (Qiagen, Hilden, Germany). Cells were disrupted in buffer RLT, homogenized, and disrupted before adding ethanol to the lysates to create conditions that subsequently promoted the selective binding of RNA to the RNeasy membrane. A total of 100 μl of sample lysate was added to a RNeasy Mini spin column, with total RNA binding to the membrane. High-quality RNA was eluted using RNase-free water. Centrifugation in a micro-centrifuge was used during all steps (binding, washing, elution).

##### Quantitative Determination by RT-PCR

Bax and Bcl-2 expression was investigated by real-time polymerase chain reaction (RT-PCR) using the BIORAD iScript^TM^ One-Step RT-PCR Kit with SYBR^®^ Green (Bio-Rad, Hercules, CA) according to the manufacturer’s instructions and as described by Labib et al. [97]. The RT-PCR reactions were performed using the following primers for the BAX, BCL-2, and β-actin genes: Bax F, 5′-GTTTCA TCC AGG ATC GAG CAG-3′; Bax R, 5′-CATCTT CTT CCA GAT GGT GA-3′; Bcl-2 F, 5′-CCTGTG GAT GAC TGA GTA CC-3′; Bcl-2 R, 5′-GAGACA GCC AGG AGA AAT CA-3′; β-actin F, 5′-GTGACATCCACACCCAGAGG-3′; and β-actin R, 5′-ACAGGATGTCAAAACTGCCC-3′. The reaction and amplifications protocol done according to Labib et al. [97]. A reaction mix (50 μL) was used, prepared as following: 2X Sybr Green RT-PCR Master (25 μl), forward primer—10 μM (1.5 10 μL), Reverse primer—10 μM (1.5 μL), nuclease-free H2O (11 μl), RNA template (1 pg to 100 ng total RNA) (10 μL), and iScript Reverse Transcriptase for One-Step RT-PCR (1 μL). The amplification protocol was performed as follows: cDNA synthesis: 50 °C (10 min), iScript Reverse transcriptase inactivation (95 °C, 5 min), PCR cycling and detection (40 cycle) (95 °C, 10 s), data collection step (60 °C, 30 s), melt curve analysis (95 °C, 1 min, 55 °C (1 min) and 55 °C (10 s) (80 cycles, increased 0.5 °C for each cycle). The reactions were performed in triplicate on a Rotor-Gene 3000 RT-PCR system. The data were analyzed by Rotor-Gene Series Software 1.7 (Build 87).

#### 3.2.10. BRAF Assay

The BRAF ELISA kit was used with cell lysates according to the manufacturer’s protocol (LifeSpan BioSciences “LSBio”, Seattle, WA, USA). HCT116 cells were cultured at density of 1 × 10^6^ and incubated for 48 h at 125, 250, 500, and 1000 µl (for MSNs and MSNsP) or 0.4, 2, 10, and 50 µg/mL (for MSNsPCOL, MSNsPCOL/CG-FA, COL, and 5-FU). The cells were then collected and pelleted by centrifugation to remove the supernatant before washing three times with PBS. Next, the cells were resuspended in PBS, lysed, and centrifuged at 1500× *g* for 10 min with cooling centrifugation to remove cellular debris. The supernatant was collected for assay, 100 µL added to the plate reader and incubated for 90 min at 37 °C, and the liquid removed. Subsequently, 100 μL of 1× Biotinylated Detection Antibody was added to each well and incubated for 1 h at 37 °C, followed by removal of the liquid and washing three times with wash buffer. Next, 100 μL of 1× HRP conjugate working solution was added to each well and incubated for 30 min at 37 °C, then replaced with 90 μL of TMB substrate solution and incubated for 15 min at 37 °C. Finally, 50 μL of stop solution was added and the absorbance measured at 450 nm using the microplate reader ROBONIK P2000 (Robonik India PVT LTD, Thane, India). Measurements were made in triplicate.

#### 3.2.11. Cytochrome c Assay

The cytochrome c was measured using the human cytochrome c ELISA kit in cell lysates according to the manufacturer’s protocol (Abcam, Austria). HCT116 cells were cultured at density of 1.2–1.8 × 10,000 cells/well and incubated for 72 h at 500 µL (for MSNs and MSNsP) or 60 µg/mL (for MSNsPCOL, MSNsPCOL/CG-FA, COL, and 5-FU). The cells were then collected and pelleted by centrifugation to remove the supernatant before washing with PBS. Next, several steps were procced as the kit protocol. Finally, absorbance was measured at 450 nm using the microplate reader (Robonik India PVT LTD, Thane, India). Measurements were made in triplicate.

#### 3.2.12. Measurement of Mitochondrial Membrane Potential

The mitochondrial membrane potential was done by means of flow cytometry with cell lysates according to the manufacturer’s protocol TMRE mitochondrial potential assay (Cyman chemical, Ann Arbor, MI, USA). HCT116 cells were cultured at density of 1.2–1.8 × 10,000 cells/well and incubated for 72 h at 500 µL (for MSNs and MSNsP) or 60 µg/mL (for MSNsPCOL, MSNsPCOL/CG-FA, COL, and 5-FU) with volume of 100 µg/mL. The cells were then collected and pelleted by centrifugation to remove the supernatant before washing with PBS. Next, we resuspended it in 100 µL of assay buffer assay, followed by adding 100 µL of TMRE buffer, and incubated for 30 min. Then, it was centrifuged, and resuspended in assay buffer, and the data collected by flow cytometry (FACSCalibur, Becton Dickinson, Franklin Lakes, NJ, USA). Finally, measurements were made in triplicate.

#### 3.2.13. Assays for MALAT-1, mir-205, Ang-2-CD44, and PD-1

##### Cell Culture and Treatment

HCT116 colon cancer cells were obtained from American Type Culture Collection (ATCC^®^ CCL-247), cultured in 96-well plates (cells density 1.2–1.8 × 10,000 cells/well) in RPMI 1640 medium supplemented with 10% FBS, 10 μg/mL of insulin, and 1% penicillin-streptomycin, and allowed to attach and grow for 24 h. The cell culture was treated with different samples, and control cells were left untreated. To prepare cell culture supernatants, after incubation, cells were harvested after detaching with trypsin and lysates collected by centrifugation. The cells were lysed with cell lysis buffer and centrifuged at 1500g for 10 min at 2–8 °C to exclude cell debris. The expression levels of angiopoietin-2 (Ang-2), PD-1, CD44, metastasis-associated lung adenocarcinoma transcript 1 (MALAT1, and miR-205 were measured in the prepared cell culture supernatants by RT-PCR or ELISA.

The supernatant was used to measure Ang-2 and PD-1 by ELISA using an ELISA plate reader (Model stat fax 2100, Awareness, Ramsey, MN, USA) according to the manufacturer’s instructions. For quantitative detection of total soluble human CD44, normal and variant isoforms were measured by ELISA. For measurement of long non-coding RNAs (lncRNAs) for MALAT1 and mir-205, cells were collected, and RNA extracted using the miRNeasy extraction kit. Total RNA including non-coding RNAs was extracted from supernatants using the miRNeasy extraction kit (Qiagen, Valencia CA, USA) and QIAzollysis reagent according to the manufacturer’s instructions. The concentration of RNA was determined using NanoDrop2000, which is very accurate for measuring even the smallest quantities of RNA (NanoDrop2000, Thermo Scientific, Wilmington, NC, USA). Reverse transcription was carried out on extracted RNA in a final volume of 20 μL using the RT2 First Strand kit (Qiagen) according to the manufacturer’s instructions. The expression levels of the studied lncRNAs were evaluated using GAPDH, which is widely used as an internal control for serum lncRNAs in numerous studies [98,99] according to the manufacturer’s protocol. The MALAT1 Ref Seq no. was NR 002819.2. The primer sequences for GAPDH were 5’-CCCTTCATTGACCTCAACTA-3’ (forward) and 5’-TGGAAGATGGTGAT GGGATT-3’ (reverse). RT-PCR was done in a 20 Μl reaction mixture using the Rotor gene Q System (ROTOR-Gene Q, SN R1211164, Qiagen, Hilden, Germany) with the following conditions: 95 °C for 10 min, followed by 45 cycles at 9 °C for 15 s and 60 °C for 60 s. The cycle threshold (Ct) method was used to quantify target genes relative to their endogenous control. The ΔCt of microRNAs was calculated by subtracting the Ct values of SNORD 68 from miR-205. The ΔCt of lncRNAs was calculated by subtracting the Ct value of GAPDH from that of MALAT1. The fold change in miR-205 and MALAT1 expression levels were calculated using the equation 2^−ΔΔCt^. Gene expression was calculated relative to the internal control (2−Ct). The fold change was calculated using 2−Ct for relative quantitation [100].

### 3.3. Statistical Analysis

Data for biological evaluations are expressed as mean ± SD. Significance differences were calculated using the Student t-test, Mann Whitney U test, and analysis of variance (ANOVA) analysis at *p* < 0.05. All statistical calculations were performed in triplicate using computer program IBM SPSS (Statistical Package for the Social Science; IBM Corp, Armonk, NY, USA) release 22 for Microsoft Windows.

## 4. Conclusions

We successfully designed a novel DDS for COL prodrug that efficiently targets cancer cells. The DDS was fabricated by loading COL into spherical mesoporous silica nanoparticles and their subsequent modification with phosphonate groups. They were subsequently coated with chitosan-glycine complex conjugated to folic acid, which acted as a targeting ligand for cancers. Full inhibition of HCT116 colon cancer cells was observed. A weaker effect was observed in HepG2 liver and PC3 prostate cancer cells. The most important characteristic of the DDS was its negligible cytotoxicity in normal cells. We observed, after 72 h of incubation with MSNsPCOL/CG-FA, low inhibition in normal BJ1 cells (4%) compared to free COL (~60%). Apoptosis (intrinsic) was found to be the main mechanism of action occurring as a consequence of the strong antimitotic effects. MSNsPCOL/CG-FA more strongly inhibited tubulin than free COL. It also increased the cell cycle at G2/M, caspase-3 activation, and Bax expression compared to COL. On the other hand, MSNsPCOL/CG-FA inhibited anti-apoptotic proteins (Bcl-2, BRAF, and CD44) more strongly than clinically used COL and 5-FU anticancer drugs. New effects of the DDS on genetic regulation and cancer related immuno-effects were found. MSNsPCOL/CG-FA significantly inhibited MALAT1, mir-205 expression, Ang-2 protein, and PD-1 compared to COL and 5-FU. We expect that the tailored DDS for COL has the potential to become a nanomedical platform for cancer treatment.

## Figures and Tables

**Table 1:** Physicochemical properties of MSNs before and after colchicine loading and polymer coating.

Sample Code	S_BET_ (m^2^/g)	Total Pore Volume ^a^ (cm^3^/g)	P, COL, CG-FA Content Calculation from Weight Loss (wt%) ^b^
MSNs	380.1	0.772	3.3
MSNsP	202.1	0.489	6.98 as P
MSNsPCOL	181.8	0.467	3.60 as COL
MSNsPCOL/CG-FA	89.5	0.352	33.48 as CG-FA

^a^ Pore volume from nitrogen adsorption-desorption measurements at 0.999 P/P°. ^b^ Calculated from the thermogravimetric analysis. MSNs: mesoporous silica nanoparticles; P: phosphate groups; COL: colchicine; CG-FA: chitosan-glycine-folic acid.

**Table 2:** Summary of the results obtained for colchicine (COL) delivery methods.

Colchicine Delivery Method	Structure	Cancer and Normal Cell Lines	Targeting Specifications
**COL (free)**	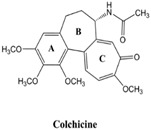	Normal cell line Cancer cell lines	High cytotoxicity (~60%) High anticancer effect at high concentration Non-targeting
**MSNsPCOL** COL-loaded silica spheres	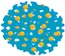	Normal cell line Cancer cell lines	Less cytotoxicity (~15%) High anticancer effect Not enough cancer targeting
**MSNsPCOL/CG-FA** COL-loaded silica spheres coated with chitosan and glycine and conjugated to folic acid	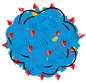	Normal cell line Cancer cell lines	Negligible cytotoxicity (~5%) High anticancer effect High cancer-targeting Specific killing of HCT116 colon cancer (achieved 100% inhibition), PC3 prostate, and HepG2 liver cells (~80% inhibition) Cancer targeting through folate receptors Enhanced apoptosis mechanism, genetic regulation, and cancer immunotherapy effects

## Supplementary Materials

The following are available online at https://www.mdpi.com/2072-6694/12/1/144/s1, Figure S1: Energy-dispersive X-ray spectroscopy analysis for elemental content (**A**), and particle size analysis by NTA in aqueous solution (**B**), Figure S2: Cell cycle analysis in HCT116 colon cancer cells: after 25 h (**A**), and after 72 h (**B**), Figure S3: Apoptosis analysis by flow cytometry measurements without and with treatments as a function of incubation time in HCT116 cells, Table S1: IC50 of MSNs before and after modification, COL loading, Coating, and folic acid conjugation after 24 h, 48 h, and 72 h incubation with cell lines.
