# Supplementary material for: Targeted Nano-Drug Delivery of Colchicine against Colon Cancer Cells by Means of Mesoporous Silica Nanoparticles
Source: Cancers (Basel). 2020 Jan 7;12(1):144. doi: 10.3390/cancers12010144 (PMC7017376; doi:10.3390/cancers12010144)
Supplement: Supplementary file 1 [file cancers-12-00144-s001.pdf]

Supplementary Materials: Targeted Nano-Drug Delivery of Colchicine Against Colon Cancer Cells by Means of Mesoporous Silica Nanoparticles

Khaled AbouAitah, Heba A. Hassan, Anna Swiderska-Sroda, Lamiaa Gohar, Olfat G. Shaker, Jacek Wojnarowicz, Agnieszka Opalinska, Julita Smalc-Koziorowska, Stanislaw Gierlotka and Witold Lojkowski

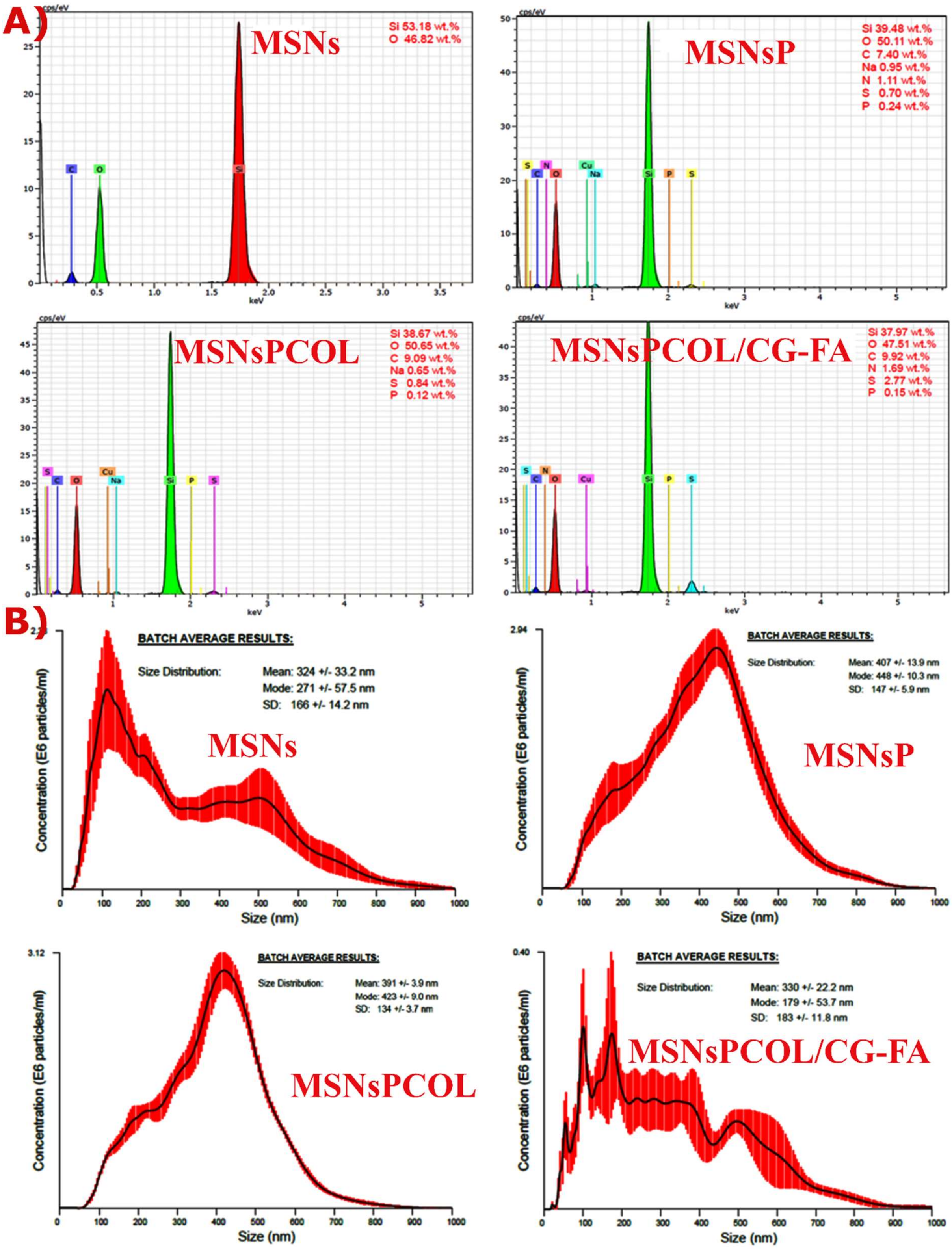

Figure 1. A. Energy-dispersive X-ray spectroscopy analysis for elemental content (**A**), and particle size analysis by NTA in aqueous solution (**B**).

Table 1. IC_{50} of MSNs before and after modification, COL loading, Coating, and folic acid conjugation after 24h, 48h and 72h incubation with cell lines.*.

		**BJ1 IC_{50}**	**HepG2 IC_{50}**	**r^2**	**PC3 IC_{50}**	**r^2**	**HCT116 IC_{50}**	**r^2**
MSNs	24h	>1000^{a}	562.8 ± 166.15^{c}	0.82	>1000^{a}		303.4 ± 71.65^{b}	0.88
	48h	>1000 ^{a}	492.9 ± 149.3^{c}	0.81	>1000^{a}		263.1 ± 65.7^{bc}	0.86
	72h	>1000 ^{a}	345.6 ± 71.9^{d}	0.89	698.6 ± 482.15^{b}	0.78	220.8 ± 72.65^{cd}	0.8
MSNs-P	24h	>1000 ^{a}	>1000a		>1000^{a}		276.9 ± 76.5^{a}	0.83
	48h	>1000 ^{a}	705.3 ± 214.1^{b}	0.84	>1000^{a}		302.0 ± 90.75^{b}	0.81
	72h	>1000 ^{a}	383.3 ± 76.3^{d}	0.90	476.4 ± 161.1^{c}	0.76	200.5 ± 31.1^{d}	0.94
MSNsP-COL	24h	>100^{b}	246.9 ± 73.95^{e}	0.91	403.9 ± 276.8^{cd}	0.78	106.5 ± 2.3^{e}	1
	48h	>100 ^{b}	207.3 ± 43.2^{ef}	0.94	336.5 ± 184.05^{de}	0.81	106.6 ± 6.25^{e}	0.99
	72h	>100 ^{b}	95.62 ± 23.38^{hi}	0.85	254.2 ± 113.75^{eg}	0.82	98.74 ± 14.6^{e}	0.94
MSNsP-COL/CG-FA	24h	>100 ^{b}	170.3 ± 36.45^{fg}	0.91	>200^{fgh}	1	19.78 ± 2.5^{fg}	0.89
	48h	>100 ^{b}	132.7 ± 32.65^{gh}	0.87	195.2 ± 15.65g^{h}	0.97	17.44 ± 13.34^{g}	0.7
	72h	>100 ^{b}	76.59 ± 12.57^{i}	0.93	124.4 ± 12.1^{h}	0.97	17.09 ± 5.08^{g}	0.91
CO pure	24h	>100 ^{b}	211.6 ± 55.15^{ef}	0.89	354.2 ± 192.35^{ce}	0.8	41.12 ± 8.82^{f}	0.89
	48h	>100 ^{b}	149.2 ± 20^{g}	0.96	299.3 ± 122.6d^{ef}	0.85	40.28 ± 7.92^{f}	0.9
	72h	>100 ^{b}	84.51 ± 25.51^{i}	0.78	144.4 ± 74.97^{h}	0.67	36.86 ± 5.93f^{g}	0.93

*Each value is expressed as mean ± standard deviation of three measurements. The statistical analysis done by one-way ANOVA at P. 0.05 based on least significant difference (LSD) according to online tool (Assaad, H.I., Zhou, L., Carroll, R.J. et al. Rapid publication-ready MS-Word tables for one-way ANOVA. SpringerPlus 3, 474 (2014) doi:10.1186/2193-1801-3-474). The difference letters indicate the significance between samples, while the same letters show no significance.

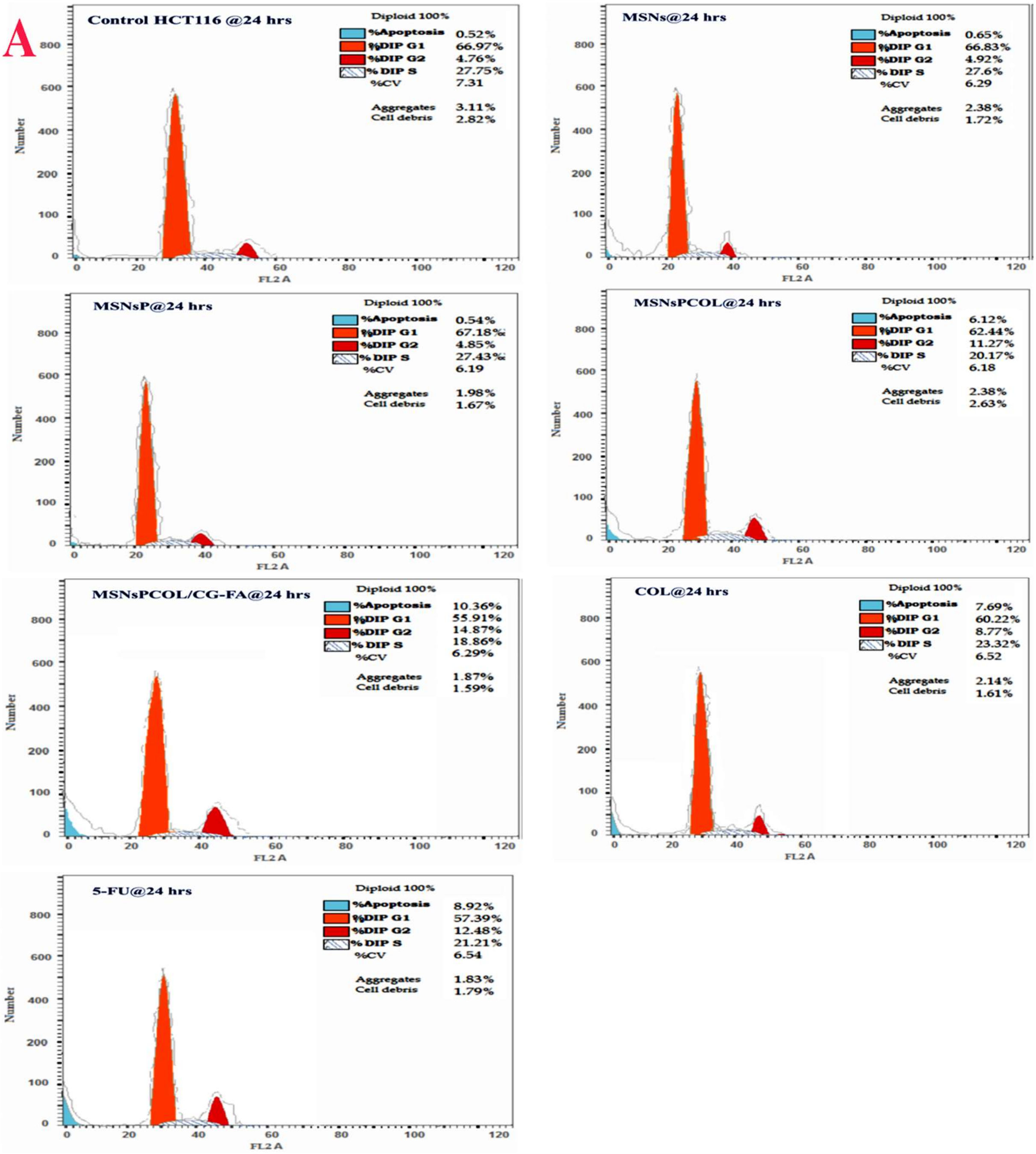

Figure S2. *Cont.*

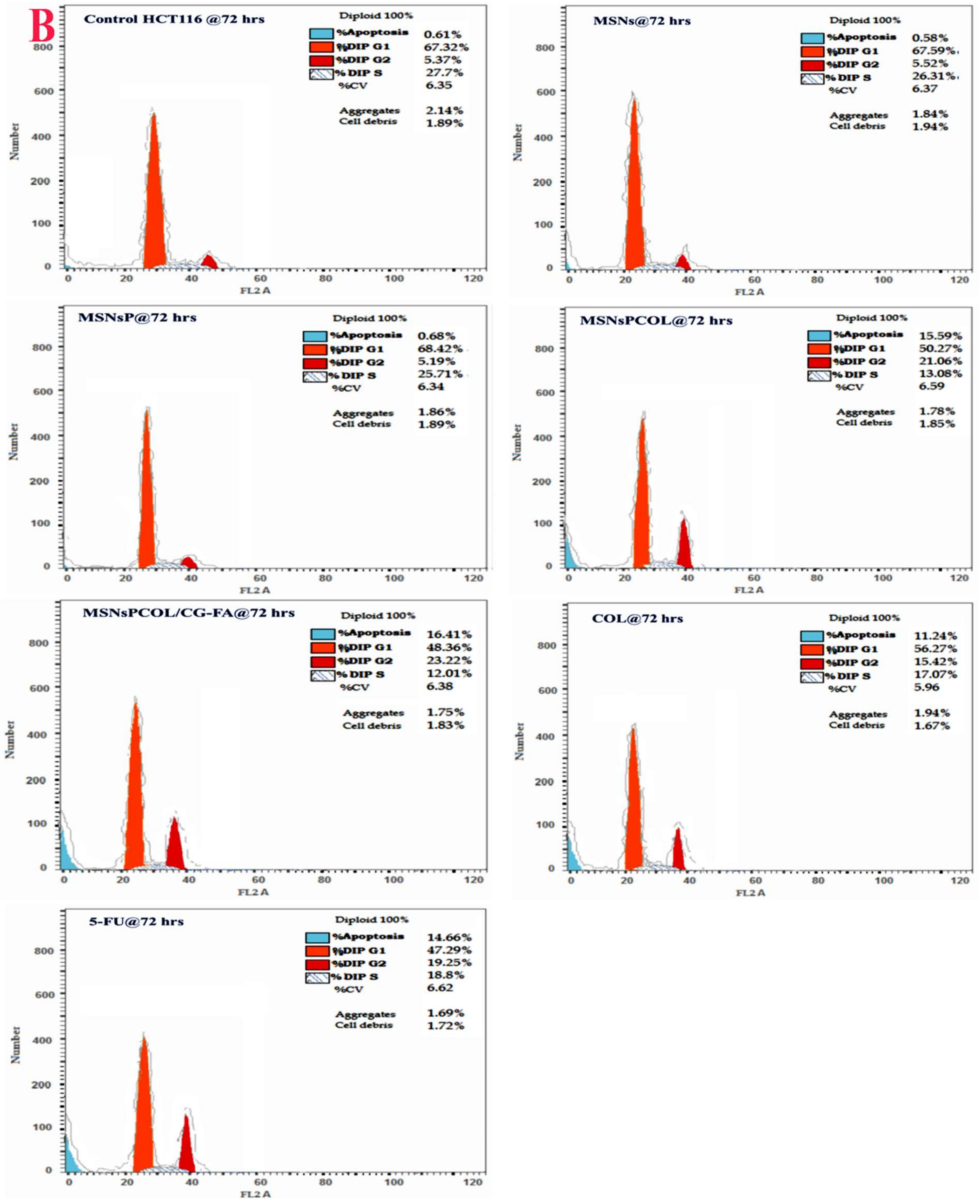

Figure S2. Cell cycle analysis in HCT116 colon cancer cells: after 25 h (**A**), and after 72 h (**B**).

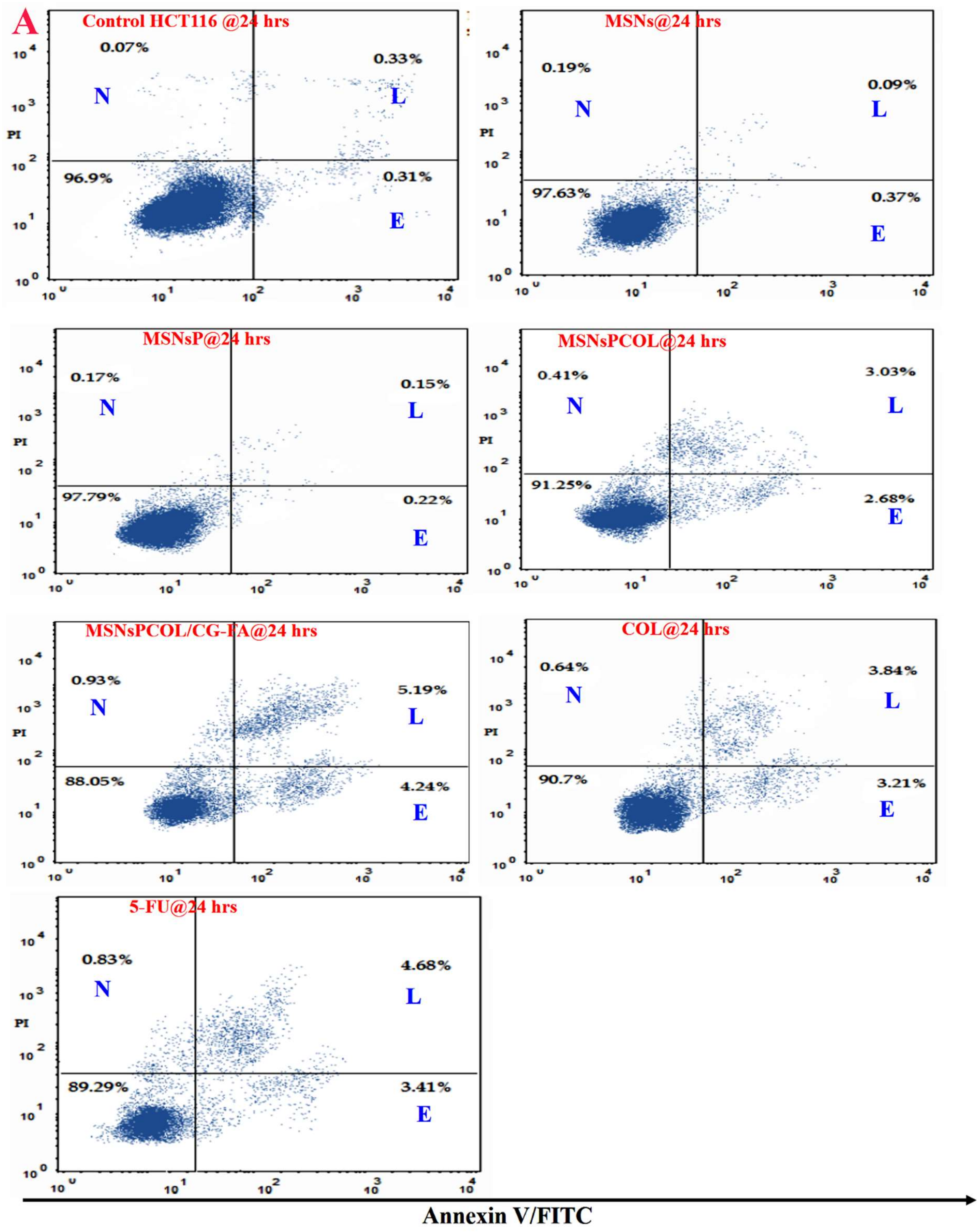

Figure S3. *Cont.*

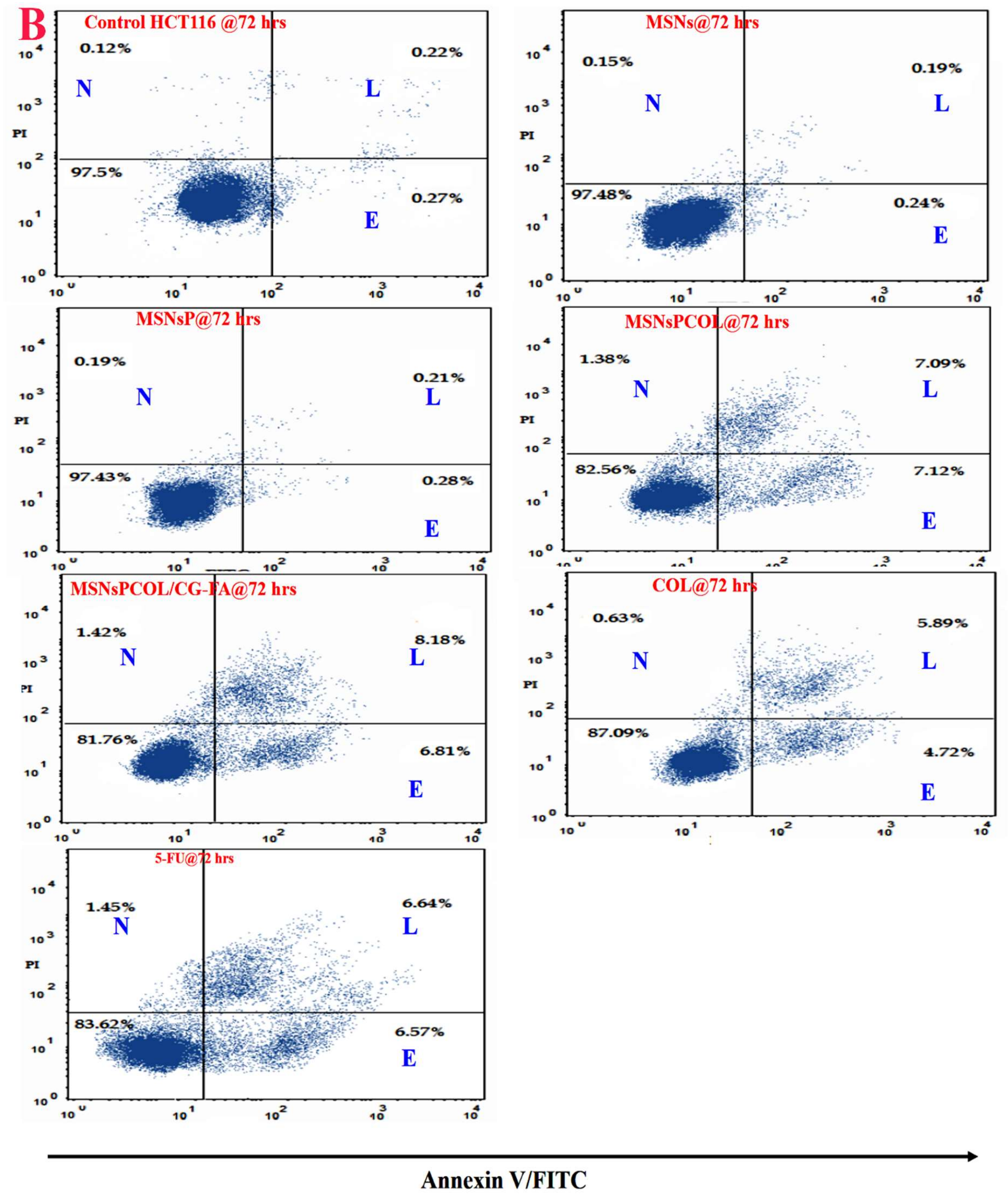

Figure 3. Apoptosis analysis by flow cytometry measurements without and with treatments as a function of incubation time in HCT116 cells. (**A**) Control cells without any treatment, and cells treated and incubated for 24 h. (**B**) Control cells without any treatment, and cells treated and incubated for 72 h. Note: E, early apoptosis; L, late apoptosis; and N, necrosis. Apoptosis evaluation in HCT116 cells was done through Annexin-V/FITC staining.

© 2020 by the authors. Licensee MDPI, Basel, Switzerland. This article is an open access article distributed under the terms and conditions of the Creative Commons Attribution (CC BY) license (http://creativecommons.org/licenses/by/4.0/).
